# TGF-beta signalling in the adult neurogenic niche promotes stem cell quiescence as well as generation of new neurons

**DOI:** 10.1111/jcmm.12298

**Published:** 2014-04-30

**Authors:** Mahesh Kandasamy, Bernadette Lehner, Sabrina Kraus, Paul Ramm Sander, Julia Marschallinger, Francisco J Rivera, Dietrich Trümbach, Uwe Ueberham, Herbert A Reitsamer, Olaf Strauss, Ulrich Bogdahn, Sebastien Couillard-Despres, Ludwig Aigner

**Affiliations:** a Institute of Molecular Regenerative Medicine, Paracelsus Medical UniversitySalzburg, Austria; b Spinal Cord Injury and Tissue Regeneration Center Salzburg, Paracelsus Medical UniversitySalzburg, Austria; c Department of Neurology, University Hospital RegensburgRegensburg, Germany; d Department of Experimental Ophthalmology, University of RegensburgRegensburg, Germany; e Institute of Biophysics and Physical Biochemistry, University of RegensburgRegensburg, Germany; f Institute of Developmental Genetics, Helmholtz Centre Munich, German Research Centre for Environmental Health (GmbH), Technical University MunichNeuherberg, Germany; g Paul Flechsig Institute for Brain Research, Department of Neuroanatomy, University of LeipzigLeipzig, Germany; h Department of Ophthalmology, SALK, Paracelsus Medical UniversitySalzburg, Austria; i Institute of Experimental Neuroregeneration, Paracelsus Medical UniversitySalzburg, Austria

**Keywords:** TGF-β1, Smad2, stem cells, cell cycle, doublecortin, differentiation

## Abstract

Members of the transforming growth factor (TGF)-β family govern a wide range of mechanisms in brain development and in the adult, in particular neuronal/glial differentiation and survival, but also cell cycle regulation and neural stem cell maintenance. This clearly created some discrepancies in the field with some studies favouring neuronal differentiation/survival of progenitors and others favouring cell cycle exit and neural stem cell quiescence/maintenance. Here, we provide a unifying hypothesis claiming that through its regulation of neural progenitor cell (NPC) proliferation, TGF-β signalling might be responsible for (*i*) maintaining stem cells in a quiescent stage, and (*ii*) promoting survival of newly generated neurons and their functional differentiation. Therefore, we performed a detailed histological analysis of TGF-β1 signalling in the hippocampal neural stem cell niche of a transgenic mouse that was previously generated to express TGF-β1 under a tetracycline regulatable Ca-Calmodulin kinase promoter. We also analysed NPC proliferation, quiescence, neuronal survival and differentiation in relation to elevated levels of TGF-β1 *in vitro* and *in vivo* conditions. Finally, we performed a gene expression profiling to identify the targets of TGF-β1 signalling in adult NPCs. The results demonstrate that TGF-β1 promotes stem cell quiescence on one side, but also neuronal survival on the other side. Thus, considering the elevated levels of TGF-β1 in ageing and neurodegenerative diseases, TGF-β1 signalling presents a molecular target for future interventions in such conditions.

## Introduction

In the adult brain, neural progenitor cells (NPCs) constantly generate new neurons in the subgranular zone (SGZ) of the hippocampal dentate gyrus (DG) and in the subventricular zone (SVZ)/olfactory bulb (SVZ-OB) system [1,2]. The cell cycle of NPCs is under tight control to avoid excessive proliferation and generation of neoplasias, to ensure the maintenance of a sufficient number of multipotent cells throughout lifetime, but also to facilitate NPC's differentiation and survival. Indeed, defects in cell cycle control mechanisms affect the stem cell pool as well as the neuronal differentiation process [3]. For example, loss of the cell cycle regulator p27 induces an expansion of the proliferating pool of progenitors but reduces neuronal differentiation [4]. Also, deletion of the cell cycle regulators p21 and ATR initiates over-proliferation of progenitors, but ultimately causes premature depletion of the NPC pool in the brain during ageing [5,6]. While profound knowledge exists on intracellular mechanisms that mediate either cell cycle control or neuronal differentiation, the extracellular signals and their immediate downstream signalling mechanisms governing cell cycle exit, and factors determining neuronal differentiation and survival are still to be fully elucidated.

Members of the transforming growth factor (TGF)-β family govern a wide range of mechanisms in brain development and in the adult, such as dorsoventral patterning, cell proliferation, neuronal/glial differentiation and survival [7]. We previously demonstrated that intracerebroventricular infusion of TGF-β1 in the adult rat brain inhibited proliferation of NPCs [8]. Similarly, in transgenic mice, overexpression of TGF-β1 in glial fibrillary acidic protein (GFAP)^+^ cells reduced proliferation of NPCs [9]. Moreover, in animal models of Huntington's disease (HD), where NPCs proliferation is reduced, we observed an enlarged pool of quiescent stem cells, which correlated with elevated TGF-β signalling in these cells [10,11]. Importantly, pharmacological blockade of TGF-β signalling in the neurogenic niche counteracts the age-associated reduction in neural stem/progenitor cell proliferation [12]. Besides inhibiting NPC proliferation and inducing stem cell quiescence, TGF-β1 promotes neuronal differentiation and survival. For example, injection of adenoviral vectors expressing TGF-β1 in the SVZ of adult rats’ brain as well as intranasal administration of TGF-β1 in adult mice after stroke increased the number of DCX-expressing immature neurons [13,14]. Moreover, TGF-β1 prevented neuronal loss in various types of acute or chronic brain damages in animal models [15–21]. Therefore, it appears that TGF-β1 might have differential effects on NPCs ranging from the induction of stem cell quiescence to the maintenance of neuronal survival.

Here, we provide a unifying hypothesis claiming that through its regulation of NPC proliferation, TGF-β signalling might be responsible for (*i*) maintaining stem cells in a quiescent stage, and (*ii*) promoting survival of newly generated neurons and their functional differentiation. Therefore, we performed a detailed histological analysis of TGF-β 1 signalling in the hippocampal neural stem cell niche of a transgenic mouse that was previously generated to express TGF-β1 under a tetracycline regulatable Ca-Calmodulin kinase promoter [18]. We also analysed NPC proliferation, quiescence, neuronal survival and differentiation in relation to elevated levels of TGF-β1 *in vitro* and *in vivo* conditions. Finally, we performed a gene expression profiling to identify the targets of TGF-β1 signalling in adult NPCs.

## Materials and methods

### Animals

Two- to three-month-old healthy female Fischer-344 rats (*N* = 5) were obtained from Charles River Laboratories (Sulzfeld, Germany). Transgenic mice expressing TGF-β1 under control of the doxycycline regulatable CamKII promoter were as previously described [18]. Induction of TGF-β1 expression in these animals was achieved by omitting doxycycline from the drinking water for 54 days (TGF-β1-on mice; *N* = 4 and TGF-β1-off mice; *N* = 4). All experiments were carried out in accordance with the European Communities Council Directive of 24 November 1986 (86/609/EEC) and were approved by the local governmental commission for animal health.

### BrdU labelling of proliferating cells

Labelling of dividing cells was performed by intraperitoneal injection of the thymidine analogue BrdU (5-bromo-2-deoxyuridine; Sigma-Aldrich, Steinheim, Germany) at 50 mg/kg of bodyweight using a sterile solution of 10 mg/ml of BrdU dissolved in a 0.9% (w/v) NaCl solution [10]. To address cell survival and cell fate, BrdU injections were performed daily on five consecutive days and mice were killed 4 weeks after the first BrdU injection.

### Tissue processing

Animals were deeply anaesthetized using ketamine (20.38 mg/ml), xylazine (5.38 mg/ml) and acepromazine (0.29 mg/ml). Transcardial perfusion was performed with 0.9% (w/v) NaCl solution, followed by 4% paraformaldehyde in 0.1 M sodium phosphate solution (pH 7.4). The brains were dissected out, post-fixed in the paraformaldehyde solution overnight at 4°C. Tissues were then cryoprotected in a 30% (w/v) sucrose in 0.1 M sodium phosphate solution (pH 7.4). Brains were cut into 40-μm-thick saggital sections using a sliding microtome on dry ice. Sections were stored at −20°C in cryoprotectant solution (ethylene glycol, glycerol, 0.1 M phosphate buffer pH 7.4, 1:1:2 by volume).

### Immunohistochemistry

Free-floating tissue sections were treated with 0.6% H_2_O_2_ in tris-buffered saline (TBS: 0.15 M NaCl, 0.1 M Tris-HCl, pH 7.5) for 30 min. Following extensive washes in TBS, sections were blocked using TBS with 0.1% Triton X-100, 1% bovine serum albumin and 0.2% teleostean gelatine (Sigma-Aldrich) for 2 hrs. The same buffer was also used for diluting the antibodies. Tissue sections were incubated with primary antibodies for overnight at 4°C. For chromogenic immunodetection, sections were washed extensively and further incubated with biotin-conjugated species-specific secondary antibodies followed by a peroxidase-avidin complex solution from Vectastain Elite ABC kit (Vector Laboratories, Burlingame, CA, USA). The peroxidase activity of immune complexes was revealed using 0.25 mg/ml 3,3′-diaminobenzidine (Vector Laboratories), 0.01% (v/v) H_2_O_2_ and 0.04% (w/v) NiCl_2_ in TBS. Tissue sections were arranged on Superfrost Plus slides (Menzel, Braunschweig, Germany) and mounted in Neo-Mount (Merck, Darmstadt, Germany). For epifluorescence immunodetection, sections were washed extensively and incubated with fluorochrome-conjugated species-specific secondary antibodies for overnight at 4°C. Sections were arranged on slides and mounted in Prolong Antifade kit (Molecular Probes, Eugene, OR, USA). Images were taken using a Leica microscope (Leica, Wetzlar, Germany) equipped with a Spot™ digital camera (Diagnostic Instrument Inc, Sterling Heights, MI, USA) and epifluorescence was observed using a confocal scanning laser microscope (Leica TCS-NT).

The following antibodies and final dilutions were used. Primary antibodies: mouse anti-TGF-bRII (1:50), rabbit anti-TGF-bRI (1:100; Santa Cruz Labs, Santa Cruz, CA, USA), rabbit anti-phospho Smad 2 (1:100) and mouse anti-Smad 2 (1:100; Cell Signalling, Danvers, MA, USA), mouse anti-PCNA (1:500) (Santa Cruz Labs), goat anti-Sox2 (1:500) (Santa Cruz Labs), guinea pig anti-GFAP (1:500; Progen, Heidelberg, Germany). Secondary antibodies: donkey antimouse, rabbit conjugated with Alexa 488 (1:1000; Molecular Probes), rhodamine X, or biotin (1:500; Jackson Immuno Research, West Grove, PA, USA). The cell nuclei were labelled with ToPro-3 (1:2000; Molecular Probes) diluted in TBS for 10 min., followed by two washing steps.

### Counting procedure

Transforming growth factor-β1 signalling was identified by the presence of pSmad2 in PCNA, GFAP, Sox2, DCX and NeuN-positive cells [10]. Immunofluorescence stainings were examined by confocal laser microscopy using a 40× PL APO oil objective (1.25 numeric aperture) and a pinhole setting that corresponded to a focal plane of 2 μm or less. For the determination of TGF-β1 signalling in neural stem cells, 50 GFAP or Sox2 or Sox2/GFAP double–positive cells were examined for pSmad2 co-localization. For the determination of TGF-β1 signalling in neurons, 50 immature or mature DCX or NeuN positive cells were examined for pSmad2 co-localization. Finally, results were represented as percentage of mean values ± SD using Prism (Prism Graph Pad Software, San Diego, CA, USA).

### NPC cultures

Neurosphere cultures of NPCs from adult hippocampus or SVZ were obtained as described previously [22]. Briefly, 2- to 3-month-old female Fischer-344 rats (Charles River Deutschland GmbH, Germany) were decapitated. Tissue was aseptically removed and dissociated. Cells were resuspended in Neurobasal (NB) medium (Gibco BRL, Eggstein, Germany) supplemented with B27 (Gibco BRL), 2 mM L-glutamine (PAN, Aidenbach, Germany), 100 U/ml penicillin/0.1 mg/l streptomycin (PAN), hereafter referred to as NB/B27. For maintenance and expansion of the cultures, the NB/B27 was further supplemented with 2 μg/ml heparin (Sigma-Aldrich), 20 ng/ml fibroblast growth factor (FGF-2) (R&D Systems, Wiesbaden-Nordenstadt, Germany) and 20 ng/ml epidermal growth factor (EGF) (R&D Systems). Cultures were maintained in T-25 culture flasks at 37°C in a humidified incubator with 5% CO_2_. Neurosphere cultures from passage number 4 to 6 were used throughout this study and termed NPCs. For passaging these cells, the culture medium containing floating neurospheres was collected in a 15-ml centrifuge tube and centrifuged at 120 × g for 5 min. The pellet was resuspended in 200 μl of Accutase (PAA, Pasching, Austria) and triturated ∼10 times using a pipette. Dissociated cells were centrifuged at 120 × g for 5 min., resuspended and reseeded.

For TGF-β1 stimulation and proliferation conditions, NPCs were seeded at a density of 5 × 10^4^ cells/ml in T75 culture flasks in NB/B27, 2 μg/ml heparin (Sigma-Aldrich), 20 ng/ml FGF-2 (R&D Systems) and 20 ng/ml EGF (R&D Systems). On the next day, TGF-β1 [R&D Systems; 10 μg/ml stock solution in 4 mM HCl with 1 mg/ml Bovine Serum Albumin (BSA)] was added to a final concentration of 10 ng/ml. Moreover, TGF-β1 was applied at the same concentration on day 4 and 7. Control cells received equal volumes of 4 mM HCl with 1 mg/ml BSA instead of TGF-β1. During the 7 days of incubation with TGF-β1, the medium was not changed.

### Immunocytochemistry

To analyse the expression of the TGF-β1 signalling components in differentiated NPCs, single cell suspensions were plated overnight on poly-ornithine (Sigma-Aldrich; 100 μg/ml) and laminin (Sigma-Aldrich; 5 μg/ml)-coated glass coverslips at a density of 10,000 cells/cm^2^. Then, 10 ng/ml TGF-β1 was added to the cells and incubated for 90 min. Finally, cells were fixed with 0.1 M phosphate-buffered 4% (w/v) paraformaldehyde (Sigma-Aldrich, Taufkirchen, Germany; 37°C, pH 7.4) for 30 min. and processed for immunofluorescence staining.

Fixed cells were washed in TBS, then blocked with solution composed of TBS, 0.1% Triton X-100 (only for intracellular antigens), 1% bovine serum albumin and 0.2% Teleostean gelatin (Sigma-Aldrich; Fish Skin Gelatine Buffer, FSGB). The same solution was used for antibody dilutions. Primary antibodies were applied overnight at 4°C. Fluorochrome-conjugated species-specific secondary antibodies were used for immunodetection. The following antibodies and final dilutions were used. Primary antibodies: rabbit or guinea pig anti-GFAP 1:1000 (Wako, Neuss, Germany); IgM mouse anti-A_2_B_5_ (1:200; Chemicon, (Millipore), Watford, Herts, UK); mouse anti-rat nestin (1:500; Pharmingen, San Diego, CA, USA). Secondary antibodies were donkey antimouse, rabbit conjugated with Alexa Fluor® 488 (Molecular Probes), rhodamine X (RHOX) 1:1000 (Dianova, Hamburg, Germany). In case of detergent-sensitive antigens, Triton X-100 was omitted from FSGB buffer. Nuclear counterstaining was performed with 4′,6′-diamidino-2-phenylindole dihydrochloride hydrate at 0.25 μg/μl (DAPI; Sigma-Aldrich). Specimens were mounted on microscope slides using Prolong Antifade kit (Molecular Probes). Epifluorescence observation and photo-documentation were realized using a Leica microscope (Leica Mikroskopie und Systeme GmbH, Germany) equipped with a Spot™ digital camera (Diagnostic Instrument Inc).

### Western blot analysis

Neural progenitor cells or tissues from different brain regions were homogenized in 0.7% NP40, 50 mM Tris-HCl (pH 8.0), 0.1 mM EDTA (pH 8.0), 250 mM NaCl, 10% glycerol, 0.2 mM Na_3_VO_4_, 1 mM PMSF, 10 mM DTT, 2 μg/ml Aprotinin and 1 μg/ml Pepstatin and centrifuged at 17,900 × g for 15 min. at 4°C. The protein concentration in the supernatants was determined using the BCA kit (Sigma-Aldrich). Protein extracts were resolved on 12% SDS-PAGE and transferred onto nitrocellulose membranes (Schleicher and Schuell, Dassel, Germany) as described previously [10]. Antibodies for Wetern blots were as follows: rabbit anti-phospho smad 2, 1:500 (Cell Signaling); mouse anti-smad 2 (1:500; Cell Signaling); mouse anti-TGFbRII (1:500; Santa Cruz Biotechnology, USA); rabbit anti-TGFbR1 (1:1000; Santa Cruz Biotechnology, Dallas, TX, USA); rabbit anti-actin (1:5000; Sigma-Aldrich). Secondary goat antimouse or anti-rabbit IgG-HRP antibodies (1:10,000; Dianova) and detection was performed with the ECL plus chemiluminescence system (Amersham/Pharmacia, Freiburg, Germany) and exposed to Hyperfilm (Amersham Pharmacia). Blots were stripped and reprobed as described [23].

### RNA preparation and microarray analyses

Total RNA was extracted from 7 days vehicle or of TGF-β1 treated cells using RNeasy Midi Kit (Qiagen, Hilden, Germany) according to the manufacturer's instructions. Biotinylated cRNA were prepared according to the standard Affymetrix protocol from total RNA (Expression Analysis Technical Manual, 2001, Affymetrix, Santa Clara, CA USA). Following fragmentation, cRNA was hybridized on GeneChip Rat Genome 230 2.0 Array (experiment 1) as well as on GeneChip Rat Expression 230A Array (experiment 2). For both experiments, GeneChips were washed and stained in the Affymetrix Fluidics Station 400 and then scanned using the Hewlett-Packard GeneArray Scanner G2500A. The expression profiling and data analysis of the two independent experiments were carried out at the competence centre for fluorescent bioanalytics (KFB; Regensburg, Germany).

### Bioinformatic analysis

Expression data of Experiment 1 and 2 were analysed with Affymetrix Gene Chip Operating Software (GCOS 1.2) and with Microarray Suite version 5.0 (MAS 5.0) using Affymetrix default analysis settings respectively. Global scaling was chosen as normalization method for each of a total of four arrays (from TGFβ1- and vehicle-treated cells of both experiments). Signals, detection *P* values and detection calls (*i.e*. ‘present’, ‘absent’ and ‘marginal’) were calculated by GCOS/MAS 5.0 algorithm for 31,099 and 15,923 probe sets of Experiment 1 on a GeneChip Rat Genome 230 2.0 Array and of Experiment 2 on a GeneChip Rat Expression 230A Array respectively. The detection call and an associated detection *P* value of a probe set (representing the corresponding transcript) was computed by statistical analysis of the probe pairs Affymetrix, 2002. In these analyses, transcripts with detection *P* < 0.04 were defined as ‘present’. Transcripts detections with 0.04 < *P* < 0.06 were defined as ‘Marginal’, and transcripts with *P* > 0.06 were called ‘absent’. In a further step, signal log ratios (SLRs), fold changes, change *P* values and change calls (*i.e*. ‘increase’, ‘marginal increase’, ‘decrease’, ‘marginal decrease’ and ‘no change’) were determined by pairwise array comparison in the GCOS/MAS 5.0 software of each experiment separately. Finally, the probe sets of experiment 1 and 2 were combined for further functional analysis regarding Gene Ontology (GO) classification. As selection criteria, we only considered transcripts that were defined as ‘present’ in at least one of the four arrays and showed differential expression values between vehicle- and TGF-β1-treated cells. Thus, a differentially expressed gene was indentified when its corresponding change call showed in case of up-regulation ‘increase’ in both experiments or ‘increase’ and ‘marginal increase’ or in case of down-regulation ‘decrease’ in either experiments or ‘decrease’ and ‘marginal decrease’. Ninety per cent of the genes obtained in this way were assigned as ‘present’ by the detection call on all four arrays. To assess the correlation of experiment 1 and 2, the SLR values of both were plotted against each other using the R statistical software (http://www.r-project.org).

To identify biological function of differentially expressed genes, significantly regulated genes were analysed with the GeneRanker software (Genomatix, Munich Germany) and mapped to GO trees. For identification of over-represented GO terms, the GeneRanker software calculated a z-score for each term. The z-score represents the difference between observed and expected annotations, and was normalized to the SD of a hypergeometric distribution. A z-score of 1.96 corresponds to a *P* value of 0.05. To account for multiple testing, a false discovery rate (fdr) was calculated for each z-score. We considered GO terms with an fdr value less than 0.19; fdr values <0.085 are considered to be statistically significant.

The gene expression data have been deposited in Gene Expression Omnibus (GEO) of NCBI [24] and are accessible by the GEO series accession numbers GSE14562 and GPL1355, as well as GSE14556 and GPL341.

### Quantitative PCR

RNA was extracted from vehicle- and TGF-β1-treated cells with the RNeasy Mini Kit (Qiagen) and cDNA was synthesized using the Reverse Transcription System (Promega, Mannheim, Germany), both according to the manufacturer's instructions. Expression analysis was validated by real-time quantitative PCR with Stratagene Mx3005P (Agilent Technologies, Waldbronn, Germany) using SYBR Green JumpStart Taq ReadyMix (Sigma-Aldrich) and specific primers for jagged 1 (forward: CTCCTGTCGGGATTTGGTTA, reverse: CTTGCCCTCGTAGTCCTCAG), doublecortin (DCX) (forward: GGAAGGGGAAAGCTATGTCTG, reverse: TTGCTGCTAGCCAAGGACTG), myelin basic protein (MBP; forward: GCTTCTTTAGCGGTGACAGG, reverse: CCAGCTAAATCTGCTGAGGG), disabled homolog 2 (Dab2; forward: CTGAAAGTGCCTTTTCTGCC, reverse: GAGCTTCCTGTTTGCCAGTC), TGF-β receptor (TGF-βR) II (forward: GGCTCCCTGAACACTACCAA, reverse: AGGGAGCAAGTCCTTGGTTT), delta-like1 (forward: ATATCTGTAGCACCGCACCC, reverse: AAATACGCCAGAGCTCCAGA), hairy and enhancer of split (HES)1 (forward: AAACCCTCAACTGCTCCGTA, reverse: GCGCCTCTTCTCCATGATAG) and achaete-scute complex homolog 1 (Mash1; forward: GGCTCAACTTCAGTGGCTTC, reverse: TGGAGTAGTTGGGGGAGATG). For quantification, standard curves were established by amplification of serial dilutions of mixed cDNA obtained from cells that were treated with vehicle or TGF-β1. Samples were assayed in triplicates and glucose-6-phosphate-dehydrogenase (G6PDH) (forward: CCAGCCTCCACAAGCACCTCAAC, reverse: AATTAGCCCCCACGACCCTCAGTA) was used as the internal control. To exclude genomic DNA contamination, a control reaction without reverse transcription (minus RT) was included. The following temperature profile was used: 95°C, 10 min.; 95°C, 30 sec. (45 cycles); annealing temperature varied dependent on the primer sequences, 1 min.; elongation: 72°C, 30 sec.

### Electrophysiological recordings

For electrophysiological recordings, vehicle and TGF-β1-treated NPCs were dissociated and seeded on poly-l-ornithine/laminin-coated coverslips in NB/B27 supplemented with heparin, EGF and FGF-2, with or without TGF-β1. The next day, electrophysiological recordings were performed. Recordings of membrane currents were performed with the whole-cell patch-clamp technique at room temperature (22–25°C). Coverslips with adherent NPCs were placed in a perfusion chamber mounted onto the stage of an inverted microscope. The cells were superfused with a standard bath solution containing: 130 mM NaCl, 3 mM KCl, 4 mM MgCl_2_, 1 mM CaCl_2_, 2.5 mM EGTA, 10 mM HEPES and 5 mM glucose, adjusted to pH 7.4 with NaOH. Studies of Na^+^ currents were performed in a K^+^-free bath solution consisting of: 125 mM NaCl, 0.5 mM CaCl_2_, 10 mM BaCl_2_, 4 mM MgCl_2_, 2.5 mM EGTA, 10 mM HEPES, 5 mM glucose adjusted to pH 7.4 with NaOH.

Patch-clamp electrodes were pulled from borosilicate glass tubes using a Zeitz DMZ Universal Puller (Zeitz, Augsburg, Germany) and showed a resistance of 3–5 MΩ. Pipettes were filled with an intracellular solution containing: 140 mM KCl, 2 mM MgCl_2_, 1 mM CaCl_2_, 2.5 mM EGTA, 10 mM HEPES (4-(2-hydroxyethyl)-1-piperazineethane sulphonic acid) and 3 mM ATP, adjusted to pH 7.4 with KOH. For studying Na^+^ currents, KCl in the pipette solution was entirely replaced by CsCl and 1 mM Ba^2+^ was added to the bath solution. TTX (100 nM) was added to the standard solution as indicated. No changes in cell size were observed during the whole-cell configuration with these solutions. All recordings were made with an HEKA EPC 10 amplifier (HEKA Electronic, Lamprecht, Germany). TIDA software (HEKA Electronic) was used for electrical stimulation as well as for data acquisition and analysis. Voltage-dependent membrane currents were elicited by an electrical stimulation protocol, which consists of a holding potential −80 mV, 10 voltage-steps of 50 ms and +10 mV increment to depolarize the cell followed by 10 voltage-steps of 50 ms and −10 mV increment to hyperpolarize the cell. The membrane capacitance and access resistance were compensated after the whole-cell configuration was established. The access resistance was compensated for values lower than 10 MΩ. The resting potential was measured directly after establishing the whole-cell configuration and before membrane capacitance or access resistance was compensated. For analysis of voltage-dependent activation, steady-state currents were plotted against the membrane potentials of the electrical stimulation. Current densities were expressed as the ratio between maximal current amplitude and whole-cell membrane capacitance (pA/pF) at given voltage depolarizations.

### Cell death analysis

#### LDH assays

Neural progenitor cells were seeded in 96-well plates and treated with vehicle or TGF-β1 for 1 week. After 7 days of incubation, the cells were transferred to 1.5 ml tubes and centrifuged at 240 × g for 5 min. 50 μl from the supernatant was mixed with equal volume of substrate mix of CytoTox 96®Non-Radioactive Cytotoxicity Assay (Promega) in a fresh 96-well plate. Cells were incubated at room temperature for 30 min. in the dark. Subsequently, 50 μl of stop solution was added to each well and the absorbance was recorded at 490 nm.

#### DNA-fragmentation assays

Cell death was further detected by measuring cytoplasmic histone-associated DNA fragments (mono- and oligonucleosomes) in the supernatant (necrosis) and lysates (apoptosis) of vehicle- and TGF-β1-treated cells using a photometric enzyme immunoassay (Cell Death Detection ELISA^PLUS^, Roche Diagnostics, Mannheim, Germany) according to the manufacturer's instructions.

Furthermore, nuclear magnetic resonance (NMR) spectroscopy was performed to investigate cell death. We had recently demonstrated that a 1.28 ppm signal in the NMR spectrum strongly correlates with the cell death of adult neural progenitors [25]. Therefore, we performed NMR spectroscopy as described [25] of 7-day TGF-β1-stimulated NPCs and quantified the presence of a 1.28 ppm signal. Briefly, ∼5 million of cells per sample were washed twice in PBS and embedded in ultralow gelling point agarose [Sigma-Aldrich; 1% agarose in PBS solution containing 10% D2O and 40 μM DSS (4,4-dimethyl-4-silapentane-1-sulfonic acid)] to avoid inhomogeneous distributions and sedimentations inside the 5 mm NMR tubes (Norell Inc., Landisville, NJ, USA). Measurements were performed at high resolution 1H-NMR Bruker Avance 600 and 800 MHz spectrometers employing a gradient-based water suppression pulse sequence 14. 64 scans with 64k datapoints and 4.7 sec. repetition time were accumulated followed by an exponential line broadening of 0.3 Hz. After Fourier transformation, the spectra were phase- and baseline-corrected manually. DSS was used as an internal reference standard (0 ppm). The quantification was obtained by deconvolution of the spectral region of interest between 1.50 and 0.82 ppm. Significance was tested as usual with two-tailed heteroscedastic Student's *t*-test (*P < 0.05; **P < 0.01; ***P < 0.001). Values are mean ± SD.

### Statistical analysis

Data are presented as mean values ± SD. Two-way anova (groups × age) and Bonferroni post-test was used for analysing numbers of PCNA and BrdU-positive cells, number of DCX-positive cells, percentage of BrdU/NeuN and of BrdU/Sox2/PCNA-positive cells. Statistical analysis was performed with Prism (Prism Graph Pad Software). The significance level was assumed at *P* < 0.05, unless otherwise indicated.

## Results

### TGF-β signalling in quiescent neural stem cells and in post-mitotic neurons of the hippocampal stem cell niche

Transforming growth factor-β receptor II (TGFbRII), receptor I (TGFbRI) and Smad2 are integral constituents of the TGF-β1 signalling cascade. Typically, ligand binding triggers phosphorylation of Smad2, and thus phospho-Smad2 (pSmad2) is widely used as an immunohistological marker for a cellular TGF-β signalling response [10,26]. We studied TGF-bRI, RII, Smad2 and pSmad2 protein expression in the hippocampus (HC), SVZ, OB, cortex (Cor) and cerebellum (CB) by Western blot analysis (Fig. S1). TGF-bRII and RI were detected in all brain regions with lowest expression in neurogenic regions HC and SVZ. Lung with highest expression of TGF-bRII served as a positive control. In contrast to the receptors’ expression, the downstream signalling molecule Smad2 was strongly expressed in different brain regions and only faintly detected in the lung. Noteworthy, in lung tissue, the presence of phosphorylated Smad2 was not detectable, *i.e*. no signs of activated form of Smad2, whereas in brain tissues, the phosphorylated form was abundant. This suggests that TGF-β signalling is active in various regions of the adult rat brain including the neurogenic regions. Also, a widespread expression of TGF-bRI and of pSmad2 in the brain was confirmed by immunohistology (Fig. S2 and S3, Table S1).

**Fig. 1:**
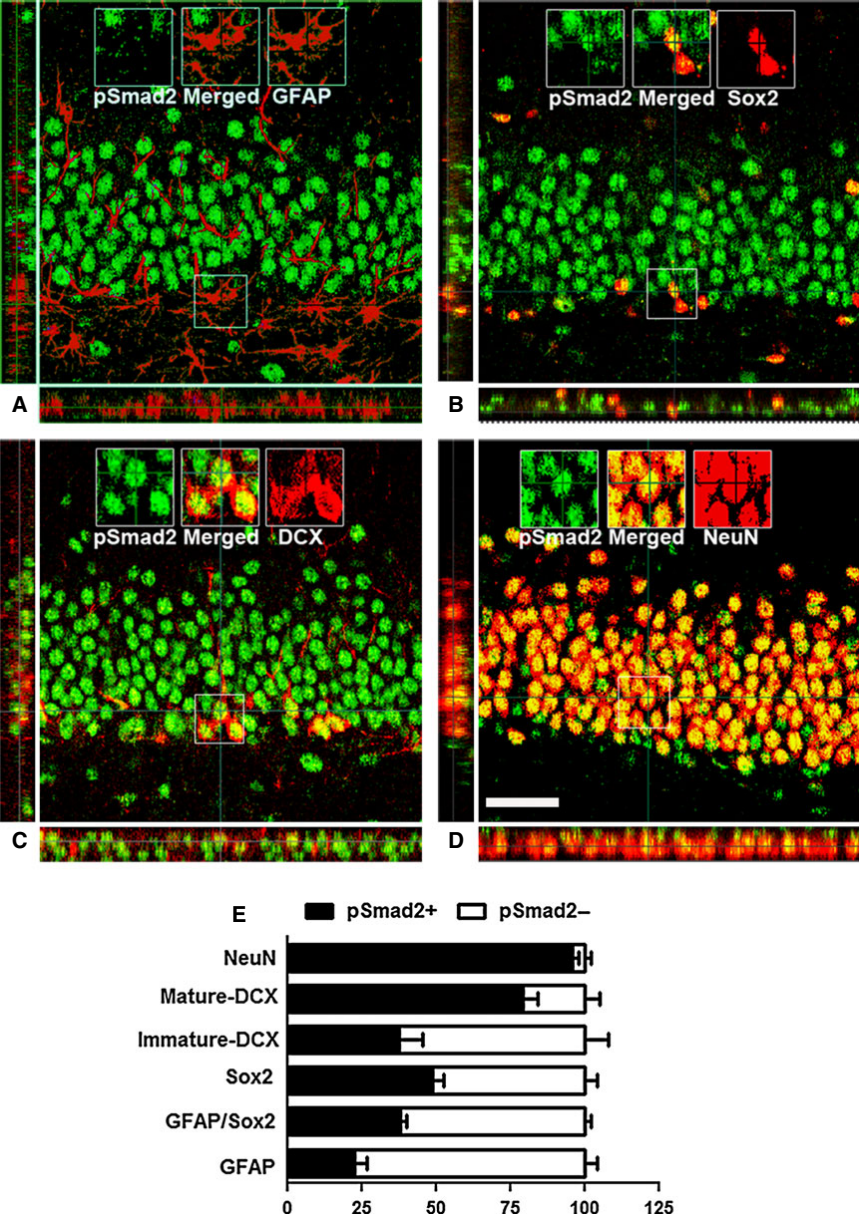
Identity of pSmad2-positive cells in the hippocampal stem cell niche. (**A**) Absence of pSmad2 (green) in glial fibrillary acidic protein (GFAP)-positive (red) cells in the subgranular zone (SGZ). (**B**) Co-localization of pSmad2 (green) in Sox2 (red)-positive cells, but not in all Sox2-positive cells in SGZ. (**C**) Appearance of pSmad2 (green) in DCX-positive cells of the hippocampal dentate gyrus (DG). (**D**) Prominant co-localization of pSmad2 (green) in NeuN (red)-positive neurons in the GCL; scale bar, 50 μm. Insets are higher magnifications of the selected fields. (**E**) Quantitative analysis of GFAP, Sox2, Sox2/GFAP double, DCX and NeuN-labelled cells that are positive for pSmad2. Note the gradual increase in percentage of cell from stem cell lineage towards neurons that co-labelled with pSmad2.

For a more detailed investigation on the expression of pSmad2 in the hippocampal neurogenic niche, we analysed neural stem cells (GFAP and/or Sox2-positive cells), young immature neurons (DCX-positive cells) and mature neurons (NeuN-positive cells) in the DG for the presence of pSmad2 immunoreactivity (Fig. 1). Most of the GFAP-positive cells within the SGZ (77.4 ± 4.1%) were devoid of pSmad2 signal (Fig. 1A and E). Likewise, the majority of GFAP/Sox2 double–positive cells (62 ± 2%) in the SGZ failed to co-localize with pSmad2 (Fig. 1E). Approximately half of the Sox2-expressing population (48.6 ± 4.1%) co-labelled with pSmad2 (Fig 1B and E). The DCX-expressing population (Fig. 1C) was classified based on their dendritic morphology [27] into immature (short horizontal processes) and mature (perpendicular dendritic arborization into the molecular layer) DCX-positive cells [27]. 37.5 ± 7.8% of DCX-positive cells with an immature morphology and 79.3 ± 5% of DCX-expressing cells with a mature morphology were positive for pSmad2 (Fig. 1E). Virtually all (96 ± 2%) NeuN-expressing cells stained for pSmad2 (Fig. 1D and E). Overall, the expression pattern of pSmad2 in the hippocampal neural stem cell niche suggests that TGF-β signalling is primarily active in cells with neuronal commitment and/or neuronal identity. Nevertheless, a small proportion of NPCs contains pSmad2 expression, suggesting a function of TGF-β1 signalling in these cells.

**Fig. 2:**
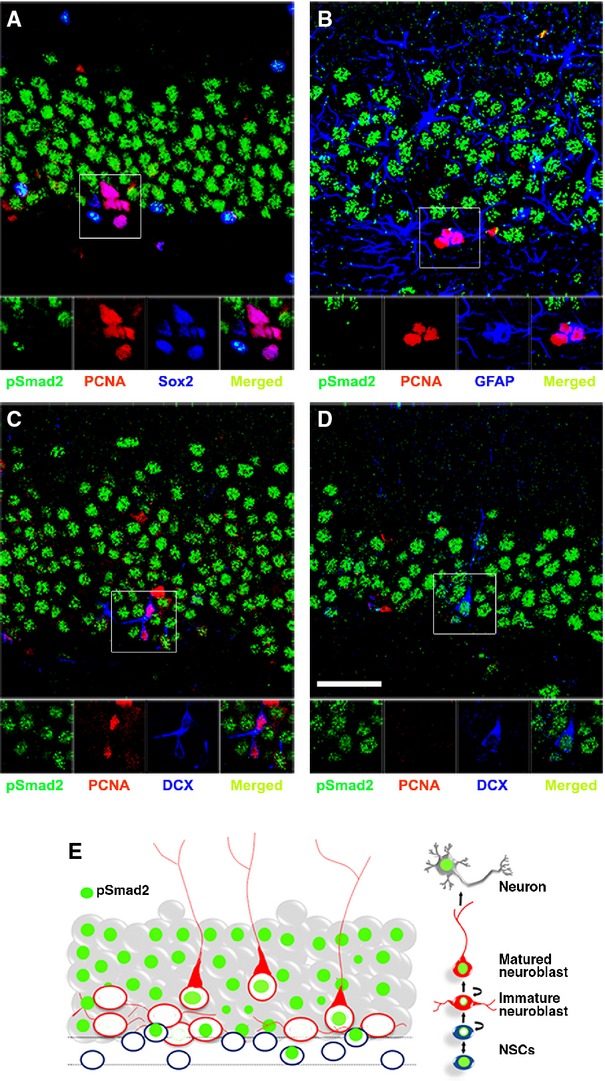
Transforming growth factor (TGF)-β1 signalling is absent in proliferating stem and progenitor cells. (**A**) Absence of pSmad2 (green) in Sox2 (blue)/PCNA (red) double–positive cells in the subgranular zone (SGZ) of the hippocampus and co-localization of pSmad2 (green) in Sox2 (blue)-positive cells that are negative for PCNA (red). (**B**) Absence of pSmad2 (green) in glial fibrillary acidic protein (GFAP; blue)/PCNA (red) double–positive cells in the SGZ of the hippocampus. (**C**) Absence of pSmad2 (green) in DCX (blue)/PCNA (red) double–positive cells in the SGZ the hippocampus. (**D**) Presence of pSmad2 (green) in DCX (blue)-positive but PCNA (red)-negative cells in the SGZ the hippocampus. Insets are higher magnifications of the selected fields; scale bar, 50 μm. (**E**) Schematic summary of pSmad2 expression (green) in neurogenesis at different cellular level in hippocampal dentate gyrus (DG). Expression of pSmad2 (green) is prominent in NeuN (grey)-positive cells and in some of the DCX (red)-expressing cells. However, it is absent in the proliferating Sox2 or GFAP or DCX-expressing cells in SGZ.

Next, we questioned if the presence of pSmad2 in cells of the SGZ and the granular cell layer (GCL) correlated with the cell's proliferative activity. Therefore, we analysed Sox2, GFAP and also DCX-expressing cells for the presence or absence of pSmad2 and proliferating cell nuclear antigen (PCNA). The expression of PCNA and pSmad2 was mutually exclusive indicating that TGF-β signalling is specific to non-proliferating cells. Sox2 and pSmad2 double–positive cells did not express PCNA in the SGZ. In contrast, the Sox2-positive/pSmad2-negative cells were positive for PCNA (Fig. 2A). Only a small proportion of GFAP-positive cells were pSmad2 positive. The only GFAP-positive cells co-labelled with PCNA were found to be pSmad2 negative (Fig. 2B). The immature DCX population was mostly negative for pSmad2, but positive for PCNA (Fig. 2C), whereas the mature DCX population was positive for pSmad2 and negative for PCNA (Fig. 2D). In summary, the proliferating neural stem and progenitor cells are mostly devoid of any detectable pSmad2, whereas quiescent NPCs as well as differentiating and mature neurons stained positive for pSmad2, indicating an active TGF-β signalling (for schematic illustration, Fig. 2E).

### Induced TGF-β1 overexpression in the hippocampus reduces cell proliferation, but promotes neuronal survival

We had recently demonstrated that 7 days of intracerebroventricular infusion of TGF-β1 reduced cell proliferation in the hippocampal DG and in the lateral ventricle wall [8]. To analyse the effects of a chronic elevation of TGF-β1 levels on the hippocampal neurogenic niche, we here used a transgenic mouse model expressing TGF-β1 in the brain under the doxycycline-controlled Ca-Calmodulin kinase promoter [18]. Withdrawal of doxycycline in this animal model results in a sustained and fourfold elevated level of TGF-β expression in the hippocampus of this animal model [18].

**Fig. 3:**
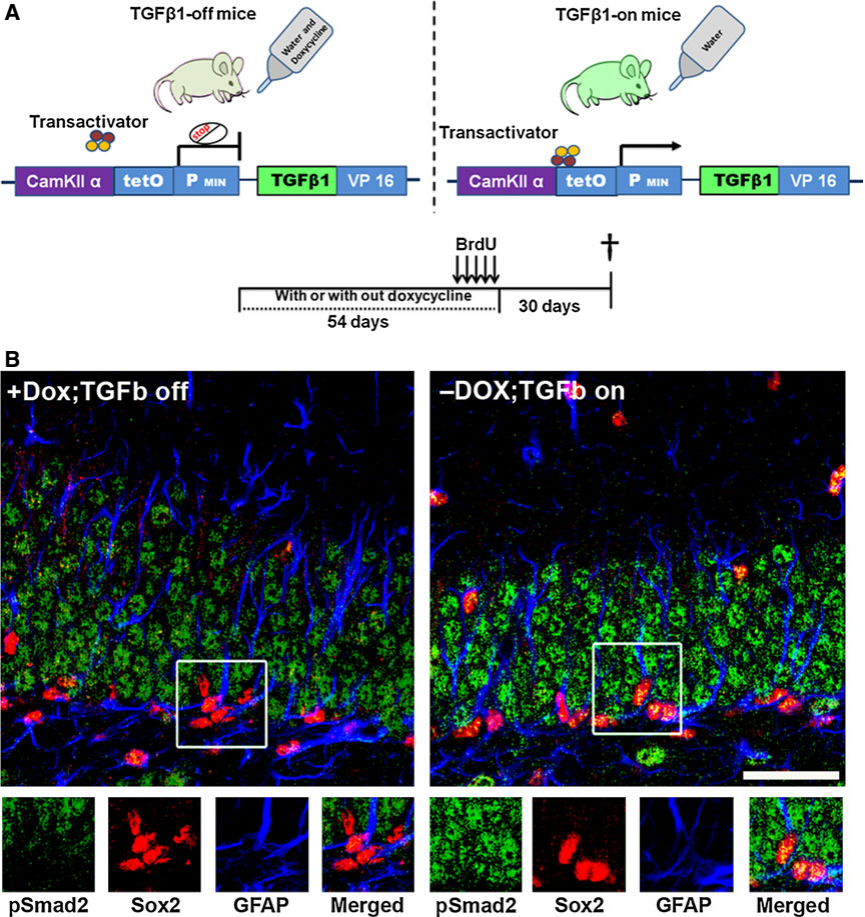
pSmad2 staining in the hippocampal dentate gyrus (DG) of transforming growth factor (TGF)-β-off and -on mice. (**A**) Schematic representation of the TGF-β1 ‘on/off’ transgenic mouse model, illustration of the doxycycline and BrdU experimental paradigms. (**B**) TGF-β1 signalling in stem cells. Note the prominent immunoreactivity of pSmad2 in the hippocampal DG of TGF-β on mice. pSmad2 (green) is strongly present in Sox2 (red)/glial fibrillary acidic protein (GFAP; blue) double–positive cells specifically in the subgranular zone (SGZ) of the hippocampus from TGF-β1-on mice; scale bar, 100 μm.

First, we analysed the pattern of pSmad2 labelling in the DG of this transgenic mouse model. A 54-day-long induction of TGF-β1 in 3-month-old transgenic mice by withdrawal of doxycycline (Fig. 3A) strongly increased the levels of pSmad2 in the DG. In addition to increasing the expression of pSmad2 in the granule cell layer, the TGF-β-on mice showed strong pSmad2 immunoreactivity in Sox2/GFAP double–positive cells, which were usually devoid of pSmad2 in the TGF-β-off animals (Fig. 3B).

**Fig. 4:**
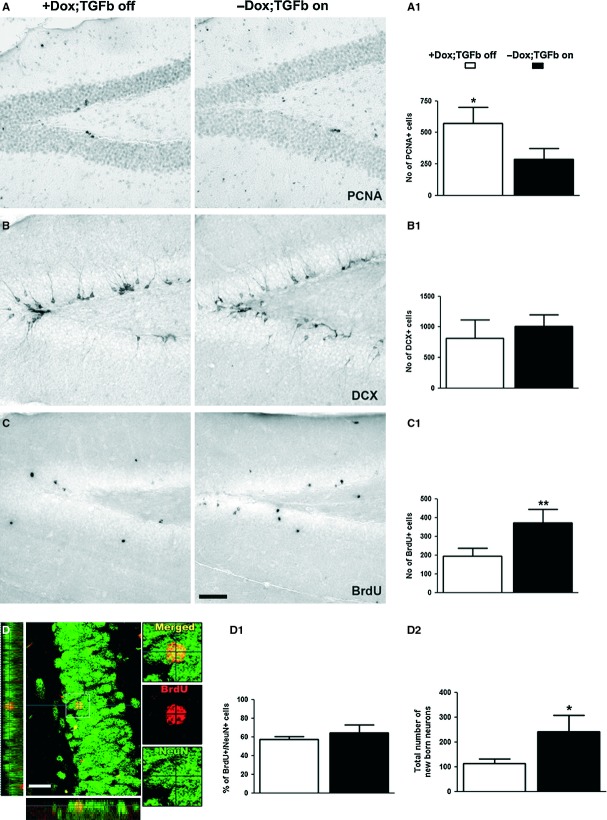
Reduced proliferation but increased neuronal survival in the hippocampus of transforming growth factor (TGF)-β-on mice. (**A-A**^**1**^) Quantification of the number of PCNA-positive cells in the dentate gyrus (DG) of TGF-β-off and TGF-β-on mice. Data are expressed as means ± SD. For statistical analysis, a Student's *t*-test was performed. Note the reduction in PCNA-positive cells in TGF-β -on mice (A^1^*, *P* < 0.05). (**B-B**^**1**^) Quantification of the number of DCX-positive cells in the DG of TGF-β-off mice and TGF-β-on mice and result are not significant (**B^1^**). (**C-C**^**1**^) Quantification of the number of BrdU-positive cells (from 4 weeks of survival experiment) in the DG of TGF-β-off mice and TGF-β-on mice. Note the increment of BrdU-positive cells in TGFβ-on mice (C^1^**, *P* < 0.01). (D-D^2^) Quantification of the phenotype of BrdU-positive cells. Note the increased total number of BrdU/NeuN double–positive neurons in the DG of TGF-β-on mice (**D**^2^*, *P* < 0.05); scale bar, 25 μm. Insets are higher magnifications of the selected fields.

The number of PCNA-positive cells indicating cell proliferation in the SGZ was significantly lower in the TGF-β-on mice compared with the TGF-β-off controls (Fig. 4A and A^1^). However, the number of young immature neurons that were positive for DCX remained unchanged (Fig. 4B and B^1^). Moreover, the number of cells surviving after 4 weeks of BrdU labelling was increased (Fig. 4C and C^1^). Thus, even though less cells were produced during TGF-β1 overexpression, the increased survival of newly generated cells resulted in a higher number of newly added cells. The fate analysis of the newly generated cells 4 weeks after BrdU incorporation revealed that ∼60%, a similar percentage of cells regardless of the treatment, of BrdU labelled had differentiated into NeuN-positive neurons (Fig. 4D and D^1^), suggesting no effect of TGF-β1 on the cell fate choice. Nevertheless, the increased survival of cells resulted in a net gain of newly born GCL neurons in the hippocampus (Fig. 4D^2^). In summary, the doxycycline-withdrawal-induced prolonged expression of TGF-β1 signalling was associated with a reduced SGZ cell proliferation, but with an enhanced survival of newly generated neurons.

### TGF-β1 signalling targets genes associated with cell cycle, cell proliferation, NPC maintenance and neuronal differentiation

**Fig. 5:**
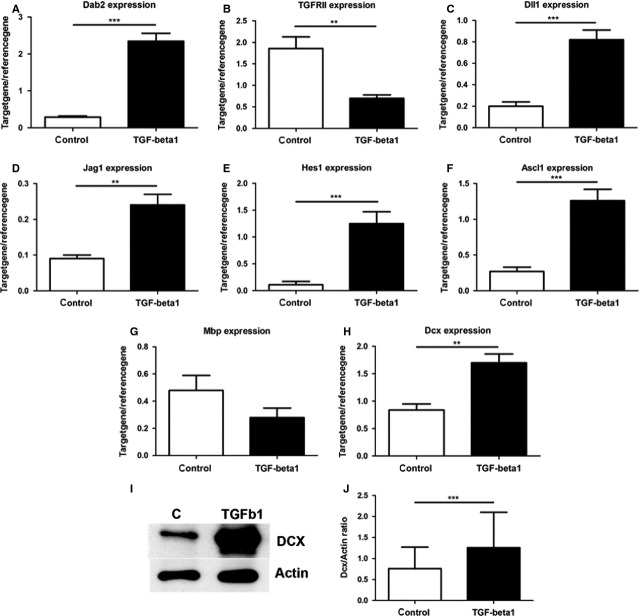
Validation of transforming growth factor (TGF)-β1 mediated transcriptome profile. (**A**–**H**) Validation of array data by quantitative RT-PCR showing increased expression of (**A**) Dab2, (**C**) Dll1, (**D**) Jag1, (**E**) Hes1, (**F**) Ascl1 (**G**) MBP and (**H**) Dcx after TGF-β1 treatment, whereas the signalling receptor (**B**) TGFRII and oligodendroglial marker (**F**) MBP were down-regulated. The quantification of the Mbp, however, revealed only a slight decrease by TGF-β1 (**F**) that did not reach statistical significance (*P* = 0.057) as demonstrated initially by DNA array analysis. (**I**) Dcx and Actin Westernblot analysis of NSC-NPC cultures incubated with or without TGF-β1 for 1 week. (**J**) Densitometric analysis of blots demonstrating induction of DCX protein by TGF-β1 as shown by an increased DCX/Actin ratio.

To substantiate the hypothesis that TGF-β signalling drives proliferating stem and progenitor cells either into stem cell quiescence or into a neuronal differentiation/survival programme, we analysed global gene expression of adult NPCs that were treated for 1 week with vehicle or with TGF-β1 under proliferation conditions, *i.e*. in the presence of EGF and FGF. Raw data of the expression analysis have been disclosed under GEO Series accession numbers GSE14562 and GPL1355, as well as GSE14556 and GPL341. Overall, 872 probe sets were significantly regulated by the TGF-β1 stimulation with 448 probe sets showing enhanced and 424 probe sets showing a reduced gene expression. This translated in 619 genes being regulated by TGF-β1 with 248 (45.9%) of them being up-regulated and 335 (54.1%) being down-regulated. Tables S2 and S3 lists the 100 genes that were the most strongly down- or up-regulated respectively. Among the regulated genes were well-known TGF-β target genes confirming that the TGF-β1 stimulation indeed induced TGF signalling. These included the TGF-β receptor adaptor disabled homolog 2 (Dab2), which is known to be induced by TGF-β and to uncouple TGF-β downstream responses from TGF-β stimulation [28] and the TGF-β RII [29]. Expression of these two genes was further validated by PCR (Fig. 5A and B).

**Fig. 6:**
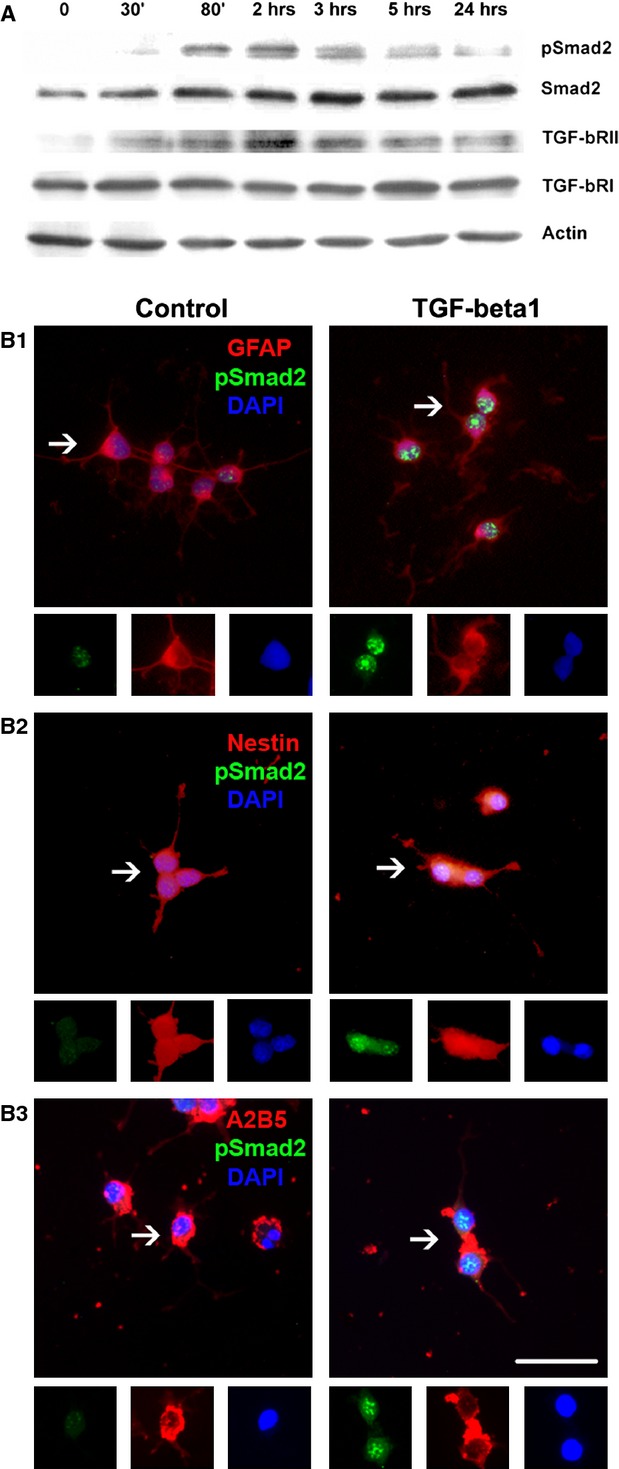
Induced transforming growth factor (TGF)-β1 signalling in stem and progenitor cells *in vitro* after TGF-β1 stimulation. (**A**) Western blot analysis of TGF-β1-treated neurosphere cultures. Note the trace amount of expressions of TGF-β RII in NPCs, in which it is slightly up-regulated at 80 min. and 2 hrs upon TGF-β1 stimulation, while expression of TGF-bRI showed a steady-state level. Smad2 is not phosphorylated in vehicle-treated neurospheres, but it peaked at 80 mins and 2 hrs upon TGF-β1 stimulation. Even though it tends to baseline, phosphorylation of pSmad2 is detectable after 12 hrs. (B1-3) Immunostaning of pSmad2 in NPCs *in vitro* with or without TGF-β1 treatment. (B1) Note the enhanced immunoreactivity and the translocation of pSmad2 (green) into the nucleus (blue-DAPI) (A) of glial fibrillary acidic protein (GFAP)-positive cells (red), (B2) of Nestin-positive cells (red) and (B3) of A_2_B_5_ cells (red) after TGF-β1 treatment, while control conditions showed only a trace amount of pSmad2 expression; scale bar, 50 μm. Insets are higher magnifications of arrowed fields.

As expected, a pathway-related analysis of the array data revealed R-Smads as the most relevant downstream key molecules. This indicates that the Smad cascade is responsible for TGF-β1-induced changes in gene expression (Fig. S4). It also confirms that NPCs responded to TGF-β1 with further activating the appropriate downstream signalling cascade. TGF-β1 stimulation indeed caused the transient phosphorylation of Smad2 as demonstrated by Western blotting (Fig. 6A). pSmad2 was below the level of detection in control neurosphere homogenates, but induced with a peak level of phosphorylation at 2 hrs after TGF-β1 stimulation (Fig. 6A). To analyse the identity of cells that responded to TGF-β1 in the NPC cultures, immunolocalization of pSmad2 in different population of NPCs was performed. This demonstrated that GFAP, Nestin, as well as A_2_B_5_-expressing populations of NPCs responded to 90 min. exposure to TGF-β1 with an increased pSmad2 staining intensity and with a strong nuclear localization (Fig. 6B1-3).

The functional assignments using the GO category ‘biological function’ illustrated that the TGF-β1 treatment induced changes in gene expression in a broad range of categories including biosynthesis and metabolism, cell proliferation, cell growth and cell cycle regulation, cell death and apoptosis, central nervous system development, neuronal maturation and synaptic transmission (Table S4–S12).

We identified many cell cycle- and cell proliferation-associated genes to be regulated by TGF-β1 (Tables S5–S8). These observations are consistent with our previous finding that a 1-week stimulation of NPC cultures with TGF-β1 under proliferation conditions inhibits cell proliferation and promotes cell cycle exit [8,10]. Among the genes identified in this study were the cell cycle regulators cyclin G1, E, D2 and B1, the known TGF-β1 target cyclin-dependent kinase inhibitor 1C (p57), the cyclin division cycle 20 homolog (cdc20), the tumour suppressor gene p53, the polo-like kinase 1 (Plk1), the cell cycle associated gene quiescin Q6 (QSCN6), suppressor of cytokine signalling 2 (Socs2), the myelocytomatosis viral oncogene homolog (Myc) and the mitogen activated protein kinase 3 (Mapk3/Erk1). A pathway-related analysis of the array data identified Mek1 (Map2k1) as a highly relevant upstream key molecules further supporting that TGF-β1 affects pathways that regulate cell proliferation (Fig. S5).

Transforming growth factor-β1 stimulation increased the expression of genes that were recently associated with NPCs and neurogenesis. Among these are the bHLH transcription factor acheate-scute complex homolog-like 1 (Mash1), the Notch signalling–associated molecules HES1, the Notch ligands delta-like 1 (dll1) and jagged1 (Jag1), the neuroectodermal progenitor marker Nestin and the bone morphogenetic protein receptor (BMPR) 1A (Tables S3, S11, S12). Here, the up-regulation of the Notch ligands dll1, Jag1 and Notch downstream mediator Hes1 is of particular interest as the elevated Notch signalling has been implicated in NPC maintenance [30]. Elevated expression of dll1 (Fig. 5C), Jag1 (Fig. 5D), Hes1 (Fig. 5E) and Mash1 (Fig. 5F) was confirmed by RT-PCR.

Besides genes that are implicated in regulation of cell cycle, cell proliferation and NPC maintenance, TGF-β1 induced changes in the expression of genes associated with oligodendroglial and neuronal differentiation, neuronal function and survival (Tables S9–S12). For example, the expression of the oligodendroglia-specific MBP gene was reduced (for qRT-PCR confirmation, see Fig. 5G), suggesting that TGF-β1 might reduce the potential of NPCs to differentiate into oligodendrocytes. Indeed, TGF-β1 treatment strongly reduced the intensity of the MBP staining (data not shown). In contrast to the lower expression of the MBP gene, the TGF-β stimulation induced the expression of many genes related to neuronal fate, neuronal differentiation and maturation (Tables S9–S12). Among these were the Notch ligand jagged1 (Fig. 5D), which is required for proper neuroblast migration and differentiation, the neuroblast migration and young immature neuronal marker DCX (Fig. 5H), and the neuroblast migration–associated gene tenascin R. A pathway-related analysis revealed that NeuroD is an upstream key molecule involved in TGF-β1-induced changes in gene expression (Fig. S6). Moreover, several neurotransmission-related genes including glutamate receptor subunits, sodium- and calcium-channel subunits were induced (Tables S2, S10), suggesting that the TGF-β treatment under proliferation conditions primes or predisposes NPCs to functional neuronal differentiation.

**Fig. 7:**
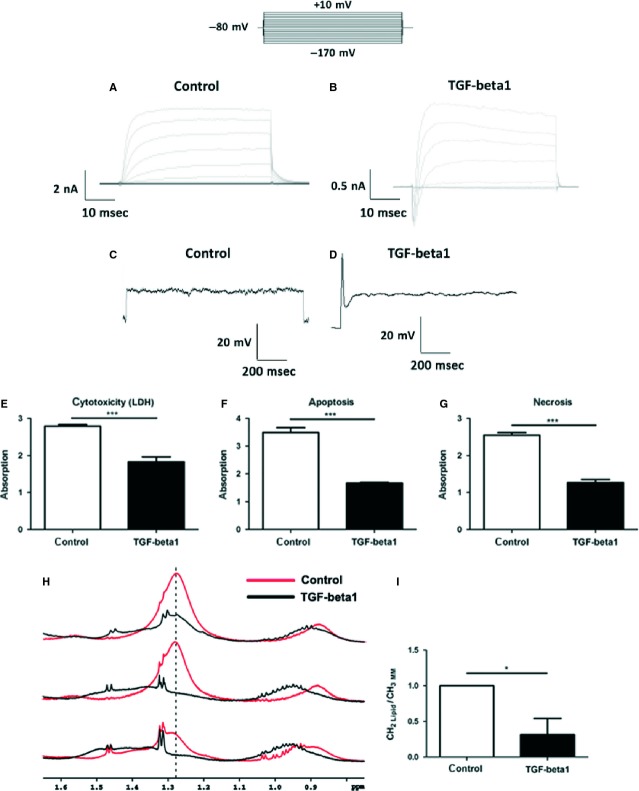
Neuronal priming and survival promoting activities of transforming growth factor (TGF)-β1 in neuropshere cultures. (**A**) Representative membrane currents incontrol NPCs. Currents were activated by an electrical stimulation protocol, which consisted of a holding potential −80 mV, 10 voltage-steps of 50 ms and +10 mV increment to depolarize the cell followed by 10 voltage-steps of 50 ms and −10 mV increment to hyperpolarize the cell. That stimulation resulted in activation of delayed-rectifier-type K^+^ channel currents (*n* = 112). (**B**) TGF-β1-treated cells (*n* = 50) responded that electrical stimulation with the same outward currents but with additional voltage-dependent fast activating and inactivating inward currents. (**C**) in current-clamp experiments current injections of 1 nA for 1s resulted in the generation of an action potential (**D**) in TGF-β1-treated cells. (**E**–**I**) Protective effect of TGF-β1 on adult rat NPCs. (**E**) TGF-β1-treated cultures contained reduced levels of LDH. In agreement, TGF-β1 limited the amount of cell death as shown by (**F**) reduced apoptosis and (**G**) necrosis in the ELISA assay. (**H**) NMR spectra of control and TGF-β1-treated NSC-NPCs. (**I**) Quantitative analysis of the 1.28 ppm fatty acid methylene signal intensity ((CH2)lip). This cell death–associated peak was integrated and normalized to cell density *via* the macromolecular methyl signals (CH3)m.m. Control cells showed a significant higher intensity of the 1.28 ppm peak indicating increased cell death without TGF-β1 treatment.

To confirm this hypothesis, we performed whole-cell patch-clamp recordings of membrane currents from NPCs that were treated for 1 week with vehicle or with TGF-β1 under proliferation conditions. Intracellular and extracellular solutions contained physiological ion compositions. Voltage-dependent membrane currents were elicited by an electrical stimulation protocol, which consists of a holding potential −80 mV, 10 voltage-steps of 50 ms and +10 mV increment to depolarize the cell followed by 10 voltage-steps of 50 ms and −10 mV increment to hyperpolarize the cell. In vehicle-treated cells, this stimulation resulted in the activation of time- and voltage-dependent outwardly rectifying currents (Fig. 7A). In a recent study, we identified these currents as currents through delayed-rectifier K^+^ channels [33]. In contrast, the same stimulation protocol activated an additional current in TGF-β1-treated cells. That depolarization-activated current is a voltage-dependent inward current with activation and inactivation and was observed before activation of the delayed rectifier (Fig. 7B). This current was identified as a current through voltage-dependent Na^+^ channels [33]. The additional expression of voltage-dependent Na^+^ channels in TGF-β1-treated cells was strong enough, so that these cells were able to develop action potentials (Fig. 7C/D). In current-clamp experiments, only TGF-β1-treated cells responded to current injections with an action potential, whereas the vehicle-treated cells did not. These action potentials were recently analysed in more detail for amplitude and kinetics [33]. Here, peak voltages at positive membrane potentials and showed a duration of 6 ms were described. Remarkably, although kept under proliferation conditions, *i.e*. presence of EGF and b-FGF, TGF-β1 induced the cells to generate sodium currents and action potentials.

Finally, to corroborate the protective effect of TGF-β1 on neural stem and progenitors, neurosphere cultures were treated for 1 week with TGF-β1 under proliferation conditions and cell death/survival was analysed by LDH, cell death ELISA and by NMR spectroscopy. We had recently demonstrated that a 1.28 ppm signal in the NMR spectrum strongly correlates with the cell death of adult neural progenitors [25]. TGF-β1-treated cultures contained reduced levels of LDH activity (Fig. 7E), showed reduced cell death in the ELISA assay (Fig. 7F) and contained a significant lower intensity of the 1.28 ppm NMR spectroscopy signal (Fig. 7H and I).

## Discussion

In this study, we illustrate that TGF-β1 signalling in the adult neurogenic niche contributes to stem cell quiescence, and to neuronal differentiation, maturation and survival of newly generated cells. This is supported by GO analyses of gene expression array data and partially by a detailed immunohistological expression analysis of the TGF-β1 downstream signalling molecule pSmad2 showing expression primarily in quiescent neural stem cells and in differentiating and mature neurons. Moreover, elevating the levels of TGF-β1 in the hippocampus in a transgenic mouse model inhibited progenitor proliferation and favoured survival of newly generated neurons. Finally, a microarray gene expression analysis of TGF-β1-stimulated NPCs supports that TGF-β1 promotes stem cell quiescence and also favours neuronal differentiation and survival. This was further underlined by experiments using neurosphere cultures, where TGF-β1 stimulation induced neuronal functionality, *i.e*. the capacity of NPCs to generate action potentials, and promoted NPCs’ survival. In general, we were focusing in the present study not on immediate but more on sustained downstream effects of TGF-β action. Therefore, for most of the *in vitro* experiments, we had chosen a 7-day TGF-β1 stimulation rather than an acute exposure.

The expression analysis of pSmad2 suggests high activity of TGF-β signalling in the brain. This supports a recent report that demonstrated an overall high level of luciferase activity in the brain of the TGF-β-responsive SMAD binding element or Smad-responsive luciferase reporter transgenic mouse model [31–33]. Indeed, TGF-β1 is present in most brain areas [34,35]. Therefore, we conclude that the healthy brain has a sustained expression of TGF-β1 that might be required for a proper brain homeostasis. Alterations in the levels of TGF-β1, either increased or decreased, might disturb brain structure and function [10,17,18,34]. Indeed, increased neuronal cell death has been observed in TGF-β1 knockout mice [36] and in mice expressing a dominant negative form of the TGF-bRIII [37]. Also, a neural-specific deletion of Smad2 in mouse brains impaired neuronal maturation, increased apoptotic cell death and displayed severe abnormalities in motor function [38]. Similarly, transgenic mice overexpressing TGF-β1 in astrocytes develop Alzheimer's disease–like pathology [39,40]. Alternatively to TGF-β1, the presence of activin in the adult brain might also contribute to the observed pSmad2 staining. Indeed, activins are present in the adult brain, are involved in the regulation of neurogenesis, in particular in neurodegeneration [41]. Here, an activin A antagonist profoundly impaired neurogenesis following the onset of kainic acid–induced neurodegeneration [41]. Nevertheless, further experiments are needed to sort out the different effects of activins and of TGF-β1 on TGF signalling in NPCs and the differential contribution of these cytokines to neurogenesis.

The gene expression array and RT-PCR analysis revealed that disabled-2 (Dab2) was one of the most induced genes after TGF-β1 stimulation. Dab2 is an adapter molecule known to maintain prolonged intracellular TGF-β signalling [28]. The Dab2 induction might explain the fact that TGF-β1-stimulated neurosphere cultures regain their full proliferative potential only 8 weeks after TGF-β1 withdrawal [8]. Hence, Dab2-coupled molecular events might be attributed to long-lasting alterations in the brain after transient alterations in TGF-β1 levels.

In the hippocampal neurogenic niche, the presence of pSmad2 was confined to quiescent neural stem cells and to differentiating and mature neurons suggesting a role for TGF-β signalling specifically in these cell populations. The percentage of Sox2 and of Sox2/GFAP-positive neural stem cells that co-label with pSmad2 might vary between animal strains. For example, in the present study, which uses Fischer 344 rats, ∼38% of Sox2/GFAP cells were positive for pSmad2, whereas in our previous work that used the wild-type littermates of transgenic HD rats, ∼20% of these cells labelled for pSmad2 [10].

Transforming growth factor-β1 treatment promoted a functional neuronal phenotype. Only these cells showed the functional expression of voltage-dependent fast activating and inactivating inward currents and, in addition, the ability to react with action potentials in current-clamp recordings in response to current injections. Although the functional ion channel expression was not analysed in full detail in this study, we were able to reproduce the same current patterns we have observed and analysed in a more detailed manner in a previous study, which used the same conditions for neuronal differentiation [33]. In that previous study, the voltage-dependent inward currents activated by depolarization could be identified as TTX-blockable Na^+^ channels currents. Furthermore, the action potentials appeared to be blockable by TTX too, reached peak voltages of +40 mV and a duration of 5 ms. Thus, it is concluded that ion properties of membrane currents and action potentials in the present study reliably reflect the functional neuronal differentiation by TGF-β1 treatment.

Elevating the levels of TGF-β *in vivo* in transgenic mice indeed further elevated the levels of pSmad2 in these cells and caused a reduction in the pool of proliferating cells and promoted the survival of newly generated neurons in the present study. The first is in line with our previous reports on the effects of infusion of exogenous TGF-β1 in the adult rat brain [8,10] and with a study analysing the effects of elevated levels of TGF-β1 in a mouse model overexpressing TGF-β1 in astrocytes [9], demonstrating impaired proliferation of NPCs and enhancement of the quiescent NPC pool [10]. Typically, levels of TGF-β1 are elevated in brains with neurodegeneration (for review, see [7,42]) and this might block NPC proliferation and neurogenesis. Vice versa, inhibiting TGF-β signalling might unlock NPCs from a TGF-β-induced cell cycle arrest. Indeed, NPCs, which are arrested in a quiescent state in brains upon irradiation or along the course of ageing – both are associated with increased expression of TGF-β1 in the neurogenic niche – can be activated to re-enter the cell cycle by pharmacological inhibition of TGF-β signalling [12]. This clearly highlights the potential to stimulate NPC proliferation by targeting TGF-β signalling with the aim to promote and sustain neurogenesis, and to counteract neurodegenerative pathologies. However, blocking TGF-β signalling might be detrimental for differentiation and survival of new neurons within the neurogenic niche, as TGF-β1 knockout mice and mice expressing a dominant negative form of the TGF-bRIII have increased neuronal cell death [36,37]. Vice versa, elevated levels of TGF-β1 promoted survival of newly generated neurons in the present study. Moreover, intranasal delivery of TGF-β1 in mice after stroke increased neurogenesis in the SVZ and adenovirally overexpressed TGF-β1 in the adult brain facilitated neuronal differentiation and sustained neuronal survival [13].

The gene expression array data highlight a pleiotropic effect of TGF-β1 on NPCs. We stimulated neurospheres with TGF-β1 under proliferation conditions, *i.e*. the presence of EGF and FGF. Under such conditions, TGF-β1 targeted the expression of genes associated with cell cycle arrest and with stem cell maintenance, as well as with neuronal fate determination, neuronal differentiation and neuronal survival. Indeed, as demonstrated in our previous work, TGF-β1 reversibly impairs NPC's proliferation [8] and promotes NPC quiescence [10]. At the same time, we demonstrate here that TGF-β1 reduces cell death in neurospheres and promotes the expression of neuronal-specific gene DCX and the electrophysiological functionality of NPCs in the presence of EGF and FGF. The latter was confirmed by a more detailed electrophysiology study demonstrating the TGF-β1 primed neural progenitors under proliferation conditions to functional maturation with firing action potentials [43]. In summary, TGF-β1 is promoting neuronal differentiation/survival as well as neural stem cell quiescence. The molecular and cellular circumstances under which TGF-β1 promotes either the one or the other require further investigation, but nevertheless, TGF-β signalling is certainly an interesting target to modulate neuroregenerative processes.

## Supporting information

**Figure S1**. Western blot analysis demonstrating TGF-β1 signalling components in intact adult brain.

**Figure S2**. Localization of TGFb-RI immunoreactivity in different areas of intact adult rat brain.

**Figure S3**. Localization of pSmad2 immunoreactivity in different areas of intact adult rat brain.

**Figure S4**. TGF-β related signalling pathway analysis.

**Figure S5**. TGF-β1 regulates expression of genes related to cell cycle of NSCs.

**Figure S6**. TGF-β1 changes the gene expression of NeuroD and related molecules in NSCs.

**Table S1**. Semi quantitative measurement of Immunoreactivity of TGF-bRII, TGF-bRI and pSmad 2 in adult rat brain.

**Table S2**. Hundred most down-regulated genes.

**Table S3**. Hundred most up-regulated genes.

**Table S4**. Gene Ontology ‘Biological Function’.

**Table S5**. TGF-β1 regulated genes ‘cell proliferation’.

**Table S6**. TGF-β1 regulated genes ‘cell growth’.

**Table S7**. TGF-β1 regulated genes ‘cell cycle’.

**Table S8**. TGF-β1 regulated genes ‘regulation of cell proliferation’.

**Table S9**. TGF-β1 regulated genes ‘neuron maturation’.

**Table S10**. TGF-β1 regulated genes ‘neuron differentiation’.

**Table S11**. TGF-β1 regulated genes ‘neurogenesis’.

**Table S12**. TGF-β1 regulated genes ‘cell fate determination’.
