# Supplementary figures and images for: TGF-beta signalling in the adult neurogenic niche promotes stem cell quiescence as well as generation of new neurons
Source: J Cell Mol Med. 2014 Apr 30;18(7):1444–59. doi: 10.1111/jcmm.12298 (PMC4124027; doi:10.1111/jcmm.12298)

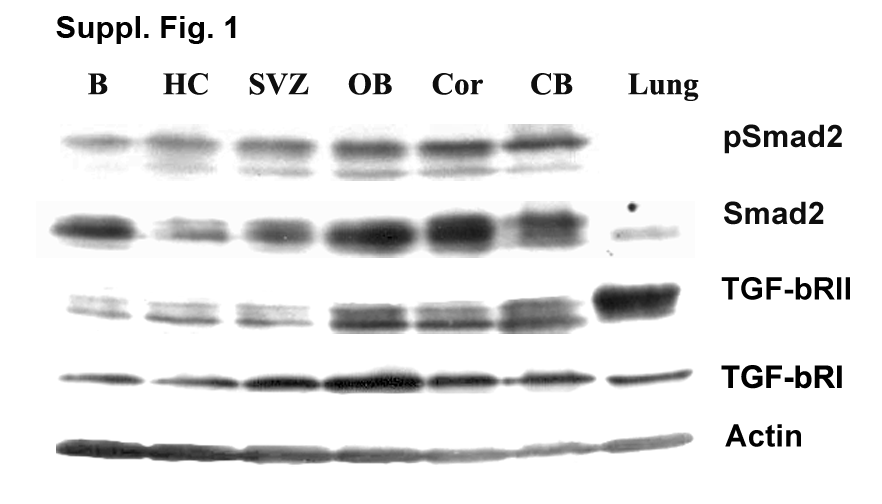

Supplement: Supplementary file 1 — Figure S1. Western blot analysis demonstrating TGF-β1 signalling components in intact adult brain. [file jcmm0018-1444-SD1.tif]

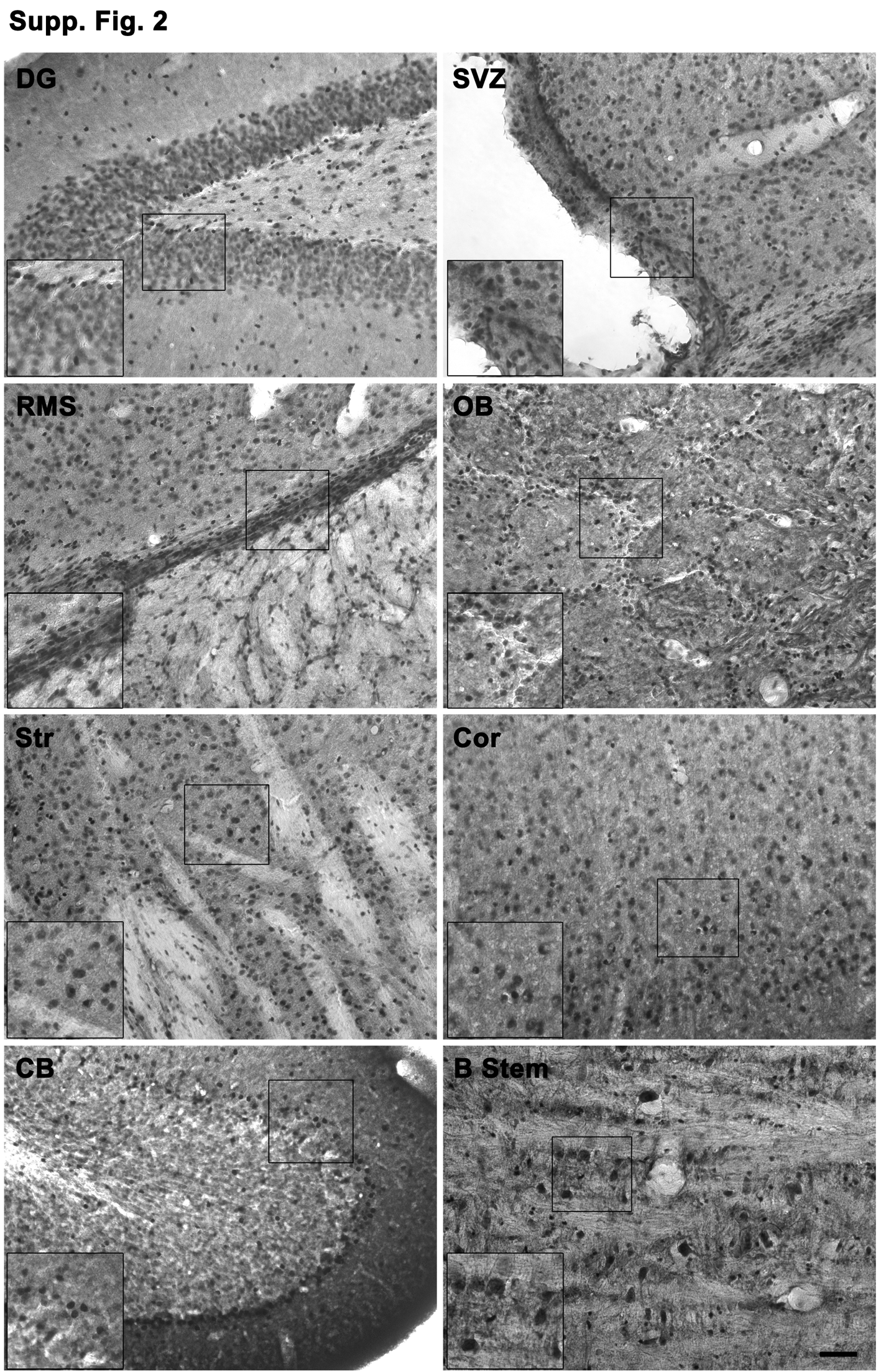

Supplement: Supplementary file 2 — Figure S2. Localization of TGFb-RI immunoreactivity in different areas of intact adult rat brain. [file jcmm0018-1444-SD2.tif]

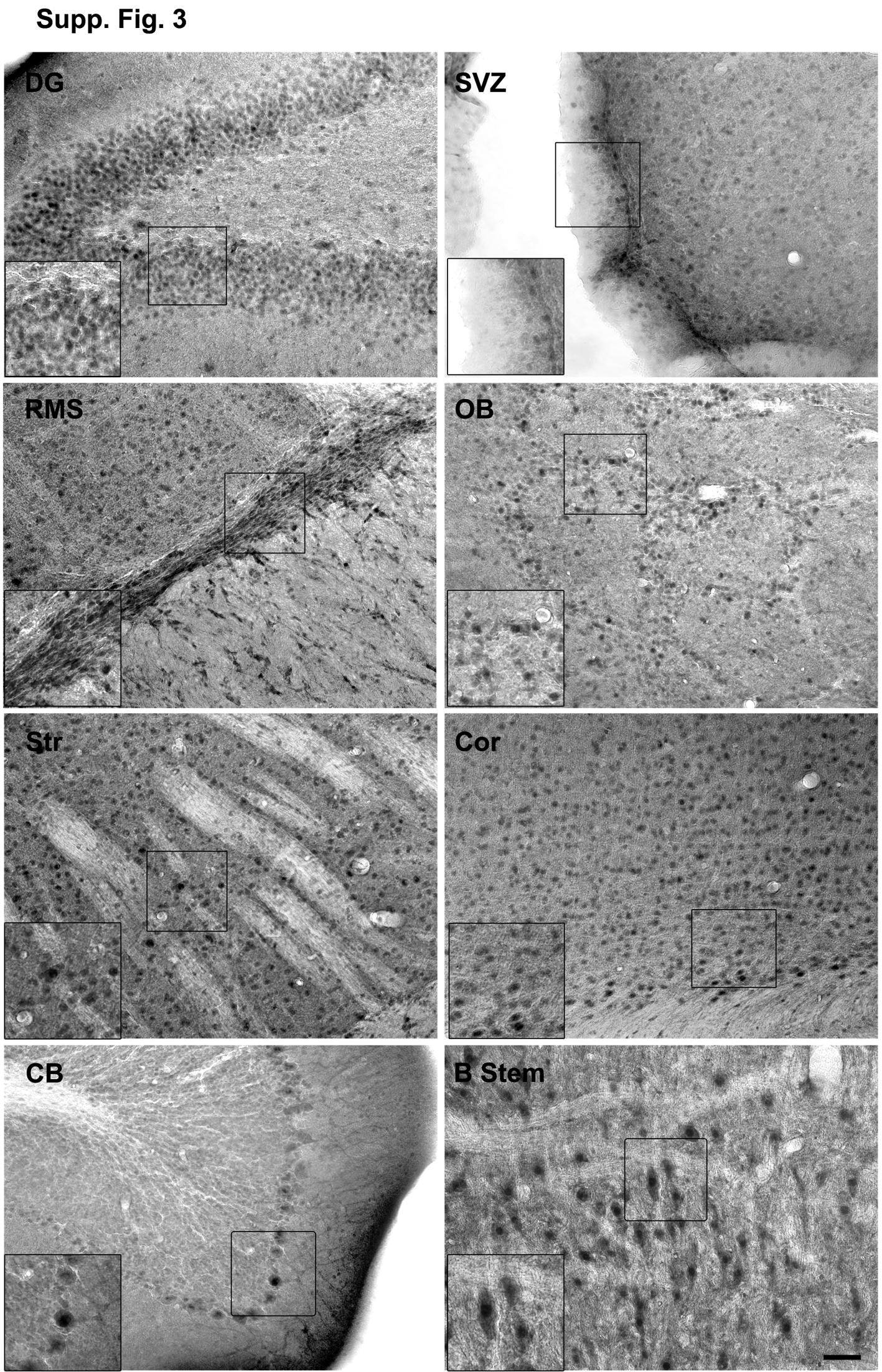

Supplement: Supplementary file 3 — Figure S3. Localization of pSmad2 immunoreactivity in different areas of intact adult rat brain. [file jcmm0018-1444-SD3.tif]

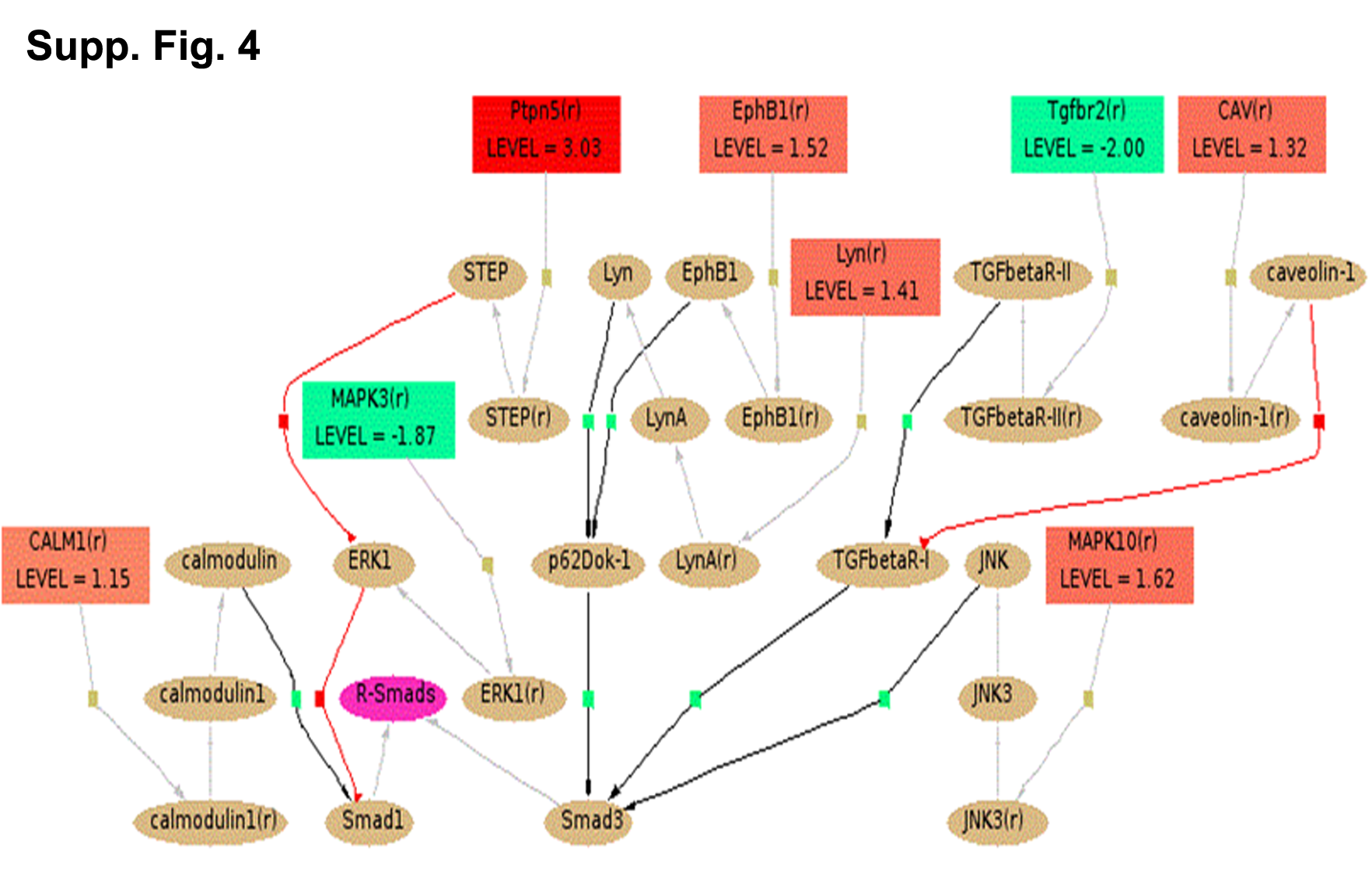

Supplement: Supplementary file 4 — Figure S4. TGF-β related signalling pathway analysis. [file jcmm0018-1444-SD4.tif]

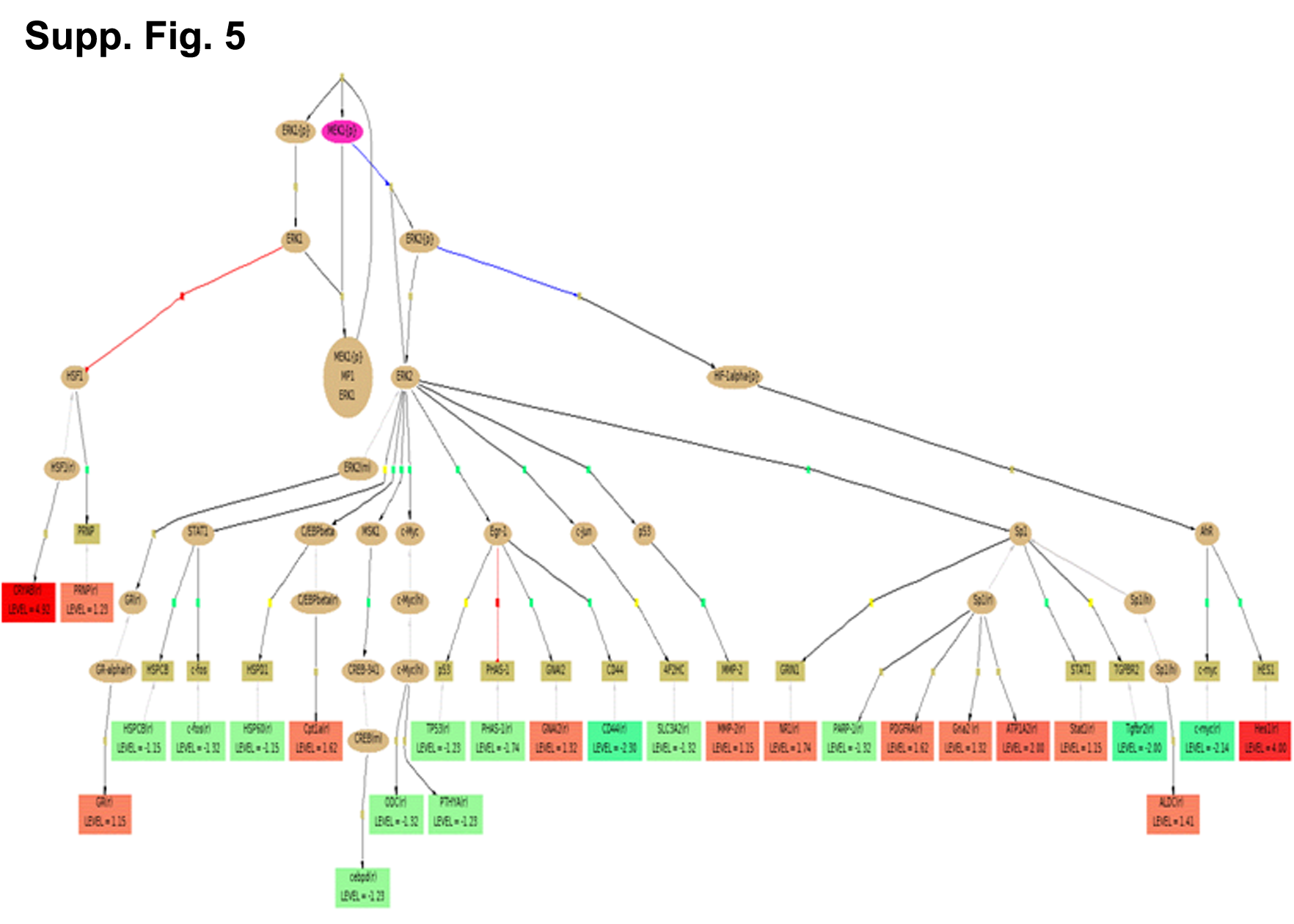

Supplement: Supplementary file 5 — Figure S5. TGF-β1 regulates expression of genes related to cell cycle of NSCs. [file jcmm0018-1444-SD5.tif]

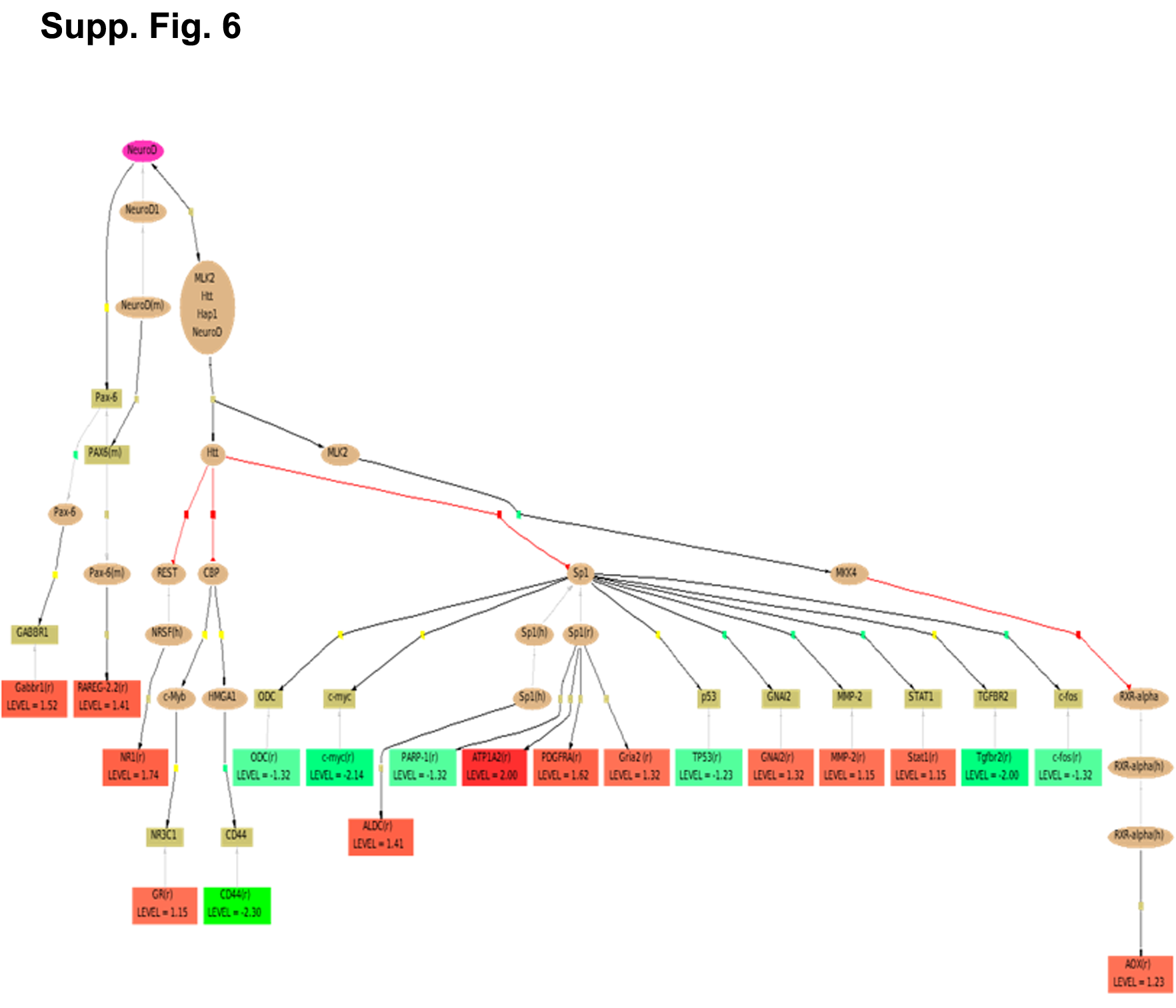

Supplement: Supplementary file 6 — Figure S6. TGF-β1 changes the gene expression of NeuroD and related molecules in NSCs. [file jcmm0018-1444-SD6.tif]
