# Supplementary material for: TGF-beta signalling in the adult neurogenic niche promotes stem cell quiescence as well as generation of new neurons
Source: J Cell Mol Med. 2014 Apr 30;18(7):1444–59. doi: 10.1111/jcmm.12298 (PMC4124027; doi:10.1111/jcmm.12298)
Supplement: Supplementary file 7 — Table S1. Semi quantitative measurement of Immunoreactivity of TGF-bRII, TGF-bRI and pSmad 2 in adult rat brain. [file jcmm0018-1444-SD7.doc]

Supplemental Table 1.

Semi quantitative measurement of Immunoreactivity of TGF-bRII, TGF-bRI and pSmad 2 in adult rat brain

| **Brain Regions** | **TGF-bRII** | | | **TGF-bRI** | **pSmad 2** |
| --- | --- | --- | --- | --- | --- |
| ***TELENCEPHALON*** | | | | | |
| **Olfactory system** | | | | | |
| Glomerular cell layer | **++** | | | **++++** | **++** |
| Granule cell layer | **+** | | | **++++** | **++++** |
| **Neocortex** | | | | | |
| Ventrolateral orbital cortex | **++** | | | **++++** | **+++** |
| Frontal cortex | **+++** | | | **++++** | **+++** |
| Parietal cortex | **+** | | | **++++** | **+++** |
| Occipital cortex | **++** | | | **++++** | **++++** |
| Entorhinal cortex | **+++** | | | **+++** | **++++** |
| **Metacortex** | | | | | |
| Cingulate/retrosplenial cortex | **+** | | | **+++** | **++++** |
| **Hippocampal formation** | | | | | |
| Dentate gyrus | **+** | | | **++** | **+++** |
| Hilus dentate gyrus | **+** | | | **+++** | **+** |
| CA1 region | **++** | | | **+++** | **+++** |
| CA2 region | **++** | | | **+++** | **+++** |
| CA3 region | **+** | | | **+++** | **+++** |
| Hippocampal fissure | **+** | | | **++** | **-** |
| Subiculum | **+** | | | **++** | **++** |
| Fimbria of hippocampus | **++** | | | **++** |  |
| Subgranular layer | **++** | | | **+++** | **++** |
| **Basal ganglia** | | | | | |
| striatum | **++** | | | **++++** | **+++** |
| Globus palidus | **+** | | | **++++** | **+** |
| Nucleus accumbens | **++** | | | **++** | **+** |
| **Amygdala** | | | | | |
| Central amygdaloid nucleus | | **++** | **+++** | | **+** |
| Medial amygdaloid nucleus | | **+** | **++** | | **+** |
| **DIENCEPHALON** | |  |  | |  |
| Thalamus | | **+** | **+++** | | **+** |
| Hypothalamus | | **++** | **++++** | | **+++** |
| **MESENCEPHALON** | |  |  | |  |
| Substantia nigra pars compacta | | **+** | **++** | | **++** |
| Substantia nigra pars reticulate | | **++** | **++** | | **+++** |
| Subthalamic nucleus | | **+** | **+++** | | **+** |
| Ventral tegmantal area | | **+** | **+++** | | **+** |
| Red nucleus | | **+++** | **+++** | | **++** |
| Superior colliculus | | **+** | **+++** | | **++** |
| Inferior colliculus | | **+** | **+++** | | **++** |
| **METENCEPHALON** | |  |  | |  |
| Pons | | **++** | **++** | | **++** |
| **Cerebellum** | |  |  | |  |
| Granular cell layer | | **+** | **++++** | | **+** |
| Purkinje cell layer | | **++** | **+++** | | **++++** |
| Deep cerebellar nuclei | | **++** | **+++** | | **++++** |
| Molecular layer | | **++** | **+** | | **+** |
| White matter | | **++** | **+** | | **-** |
| **Nonneuronal cell areas** | |  |  | |  |
| Choroid plexus | | **+++** | **++++** | | **+** |
| Ependymal cells | | **+** | **++++** | | **+++** |
| **Neurogenic areas** | |  |  | |  |
| Subventricular zone | | **+** | **++++** | | **+** |
| Subgranular zone | | **+** | **+++** | | **+** |
| Rostral Migratory Stream(RMS) | | **+** | **++++** | | **++** |

Each brain region was evaluated for the relative density of TGFbR2, TGFbR1 or pSamd2 immunostaining. – = no stained cells observed, + = only few cells/fibers stained, ++ = sparse staining (stained cells/fibers in < 25% field), +++ = strong staining (stained cells/fibers in <50% field), ++++ = robust staining (stained cells/fibers in >50% field).
