# Supplementary material for: TGF-beta signalling in the adult neurogenic niche promotes stem cell quiescence as well as generation of new neurons
Source: J Cell Mol Med. 2014 Apr 30;18(7):1444–59. doi: 10.1111/jcmm.12298 (PMC4124027; doi:10.1111/jcmm.12298)
Supplement: Supplementary file 8 — Table S2. Hundred most down-regulated genes. [file jcmm0018-1444-SD8.doc]

| **Supp. Table 2. Hundred most down-regulated genes** | |  | | |
| --- | --- | --- | --- | --- |
|  | | Fold change  (TGF-beta1 versus control) | | |
| gene title | gene symbol | experiment 1 | experiment 2 | mean |
| serine (or cysteine) proteinase inhibitor, clade B (ovalbumin), member 7 | Serpinb7 | -2,83 | -6,50 | -4,66 |
| Inhibitor of DNA binding 3, dominant negative helix-loop-helix protein | Id3 | -5,28 | -2,46 | -3,87 |
| myelocytomatosis viral oncogene homolog (avian) | Myc | -2,14 | -3,73 | -2,94 |
| selenoprotein P, plasma, 1 | Sepp1 | -1,74 | -4,29 | -3,01 |
| prostaglandin D2 synthase | Ptgds | -4,29 | -1,41 | -2,85 |
| CD44 antigen | Cd44 | -2,30 | -2,64 | -2,47 |
| asparagine synthetase | Asns | -1,87 | -2,83 | -2,35 |
| G protein-coupled receptor 37 | Gpr37 | -2,46 | -1,74 | -2,10 |
| similar to RIKEN cDNA 1810029B16 (predicted) | RGD1305222_predicted | -1,41 | -3,03 | -2,22 |
| similar to RIKEN cDNA 2310075C12 (predicted) | RGD1305625_predicted | -2,00 | -2,00 | -2,00 |
| eukaryotic translation initiation factor 4E binding protein 1 | Eif4ebp1 | -1,74 | -2,30 | -2,02 |
| nucleoporin 107 | Nup107 | -1,32 | -3,03 | -2,18 |
| Transforming growth factor, beta receptor II | Tgfbr2 | -2,00 | -1,87 | -1,93 |
| cyclin-dependent kinase inhibitor 1C (P57) | Cdkn1c | -2,00 | -1,87 | -1,93 |
| serine hydroxymethyl transferase 2 (mitochondrial) (predicted) | Shmt2_predicted | -1,62 | -2,30 | -1,96 |
| Nuclear receptor subfamily 2, group F, member 2 | Nr2f2 | -2,00 | -1,74 | -1,87 |
| similar to tetraspanin similar to TM4SF9 (predicted) | RGD1305714_predicted | -1,74 | -2,00 | -1,87 |
| Similar to RIKEN cDNA 1300002C08 gene (predicted) | --- | -1,74 | -2,00 | -1,87 |
| processing of precursor 1, ribonuclease P/MRP family, (S. cerevisiae) (predicted) | Pop1_predicted | -1,74 | -2,00 | -1,87 |
| similar to FLI-LRR associated protein-1 | LOC367314 | -1,74 | -2,00 | -1,87 |
| serine hydroxymethyl transferase 1 (soluble) (predicted) | Shmt1_predicted | -1,62 | -2,14 | -1,88 |
| nucleolar and coiled-body phosphoprotein 1 | Nolc1 | -1,52 | -2,30 | -1,91 |
| similar to Putative methyltransferase HUSSY-03 (Williams-Beuren syndrome chromosome region 22 protein homolog) /// similar to Putative methyltransferase HUSSY-03 (Williams-Beuren syndrome chromosome region 22 protein homolog) | LOC360830 /// LOC368084 | -1,52 | -2,30 | -1,91 |
| plasticity-related protein PRG-2 | RGD:727823 | -2,14 | -1,52 | -1,83 |
| actin related protein 2/3 complex, subunit 1B | Arpc1b | -1,87 | -1,74 | -1,80 |
| Suppressor of cytokine signaling 2 | Socs2 | -1,87 | -1,74 | -1,80 |
| glutamate oxaloacetate transaminase 1 | Got1 | -1,74 | -1,87 | -1,80 |
| similar to RIKEN cDNA D430044G18 (predicted) | RGD1309038_predicted | -2,00 | -1,62 | -1,81 |
| phosphoserine aminotransferase 1 | Psat1 | -1,62 | -2,00 | -1,81 |
| myosin IG (predicted) | Myo1g_predicted | -1,41 | -2,30 | -1,86 |
| inosine 5-monophosphate dehydrogenase 2 | Impdh2 | -1,41 | -2,30 | -1,86 |
| solute carrier family 7 (cationic amino acid transporter, y+ system), member 1 | Slc7a1 | -1,62 | -1,87 | -1,75 |
| 3-phosphoglycerate dehydrogenase | Phgdh | -1,62 | -1,87 | -1,75 |
| spermidine/spermine N1-acetyl transferase | Sat | -1,62 | -1,87 | -1,75 |
| nestin | Nes | -1,41 | -2,14 | -1,78 |
| Similar to RIKEN cDNA 2010309E21 (predicted) | --- | -1,41 | -2,14 | -1,78 |
| similar to hypothetical protein FLJ12442 (predicted) | RGD1305524_predicted | -1,32 | -2,30 | -1,81 |
| similar to A230072I16Rik protein (predicted) | RGD1306819_predicted | -1,23 | -2,46 | -1,85 |
| replication factor C (activator 1) 4 (predicted) | Rfc4_predicted | -1,15 | -2,64 | -1,89 |
| myelin basic protein | Mbp | -2,30 | -1,23 | -1,76 |
| tropomyosin 3, gamma /// tropomyosin isoform 6 | Tpm3 /// RGD:708368 | -1,52 | -1,87 | -1,69 |
| similar to methylenetetrahydrofolate dehydrogenase (NAD) (EC 1.5.1.15) / methenyltetrahydrofolate cyclohydrolase (EC 3.5.4.9) precursor - mouse | LOC313410 | -1,41 | -2,00 | -1,71 |
| similar to RIKEN cDNA 6720458F09 gene | MGC94251 | -1,41 | -2,00 | -1,71 |
| similar to nucleolar protein family A, member 1 | LOC499709 | -1,41 | -2,00 | -1,71 |
| Cell division cycle associated 7 (predicted) | --- | -1,32 | -2,14 | -1,73 |
| kinesin family member 20A (predicted) | Kif20a_predicted | -1,32 | -2,14 | -1,73 |
| Fibrillarin (predicted) | --- | -1,23 | -2,30 | -1,76 |
| spermidine synthase | Srm | -1,23 | -2,14 | -1,69 |
| Origin recognition complex, subunit 6-like (S. cerevisiae) (predicted) | --- | -1,23 | -2,14 | -1,69 |
| similar to RIKEN cDNA 1810014L12 (predicted) | RGD1307423_predicted | -1,23 | -2,14 | -1,69 |
| aldehyde dehydrogenase 2 | Aldh2 | -1,87 | -1,41 | -1,64 |
| nidogen 2 (predicted) | Nid2_predicted | -1,87 | -1,41 | -1,64 |
| FtsJ homolog 3 (E. coli) (predicted) | Ftsj3_predicted | -1,52 | -1,74 | -1,63 |
| isoleucine-tRNA synthetase (predicted) | Iars_predicted | -1,52 | -1,74 | -1,63 |
| matrix metalloproteinase 11 | Mmp11 | -1,41 | -1,87 | -1,64 |
| Unknown function | Bing4 | -1,41 | -1,87 | -1,64 |
| S-adenosylhomocysteine hydrolase | Ahcy | -1,32 | -2,00 | -1,66 |
| macrophage migration inhibitory factor | Mif | -1,32 | -2,00 | -1,66 |
| translocase of inner mitochondrial membrane 8 homolog a (yeast) | Timm8a | -1,32 | -2,00 | -1,66 |
| similar to hypothetical brain protein similar to X96994 BR-1 protein (Helix pomatia) | MGC95260 | -1,32 | -2,00 | -1,66 |
| polymerase (DNA directed), delta 2, regulatory subunit (predicted) | Pold2_predicted | -1,32 | -2,00 | -1,66 |
| RRS1 ribosome biogenesis regulator homolog (S. cerevisiae) (predicted) | --- | -1,32 | -2,00 | -1,66 |
| similar to ribosomal protein P0-like protein; 60S acidic ribosomal protein PO; ribosomal protein, large, P0-like (predicted) | RGD1311709_predicted | -1,32 | -2,00 | -1,66 |
| Ribosomal protein, mitochondrial, L12 | --- | -1,32 | -2,00 | -1,66 |
| Stromal cell-derived factor 2-like 1 (predicted) | --- | -1,15 | -2,30 | -1,72 |
| similar to RIKEN cDNA 2610029G23 | LOC363485 | -1,15 | -2,30 | -1,72 |
| mitogen activated protein kinase 3 | Mapk3 | -1,87 | -1,32 | -1,59 |
| hypoxanthine guanine phosphoribosyl transferase | Hprt | -1,41 | -1,74 | -1,58 |
| adenosine kinase | Adk | -1,41 | -1,74 | -1,58 |
| guanine nucleotide binding protein-like 3 (nucleolar) | Gnl3 | -1,41 | -1,74 | -1,58 |
| NMDA receptor-regulated gene 1 (predicted) | Narg1_predicted | -1,41 | -1,74 | -1,58 |
| complement component 1, q subcomponent binding protein | C1qbp | -1,32 | -1,87 | -1,59 |
| AKT1 substrate 1 (proline-rich) (predicted) | Akt1s1_predicted | -1,32 | -1,87 | -1,59 |
| nudix-type motif 5 | Nudt5 | -1,32 | -1,87 | -1,59 |
| minichromosome maintenance protein 7 | RGD:1303018 | -1,23 | -2,00 | -1,62 |
| Ly1 antibody reactive clone (predicted) | Lyar_predicted | -1,23 | -2,00 | -1,62 |
| Similar to RIKEN cDNA 2810037C03 (predicted) | --- | -1,23 | -2,00 | -1,62 |
| Eukaryotic translation initiation factor 1A (predicted) | LOC317163 | -1,23 | -2,00 | -1,62 |
| similar to hypothetical protein FLJ10156 (predicted) | RGD1308747_predicted | -1,23 | -2,00 | -1,62 |
| Synaptotagmin binding, cytoplasmic RNA interacting protein (predicted) | --- | -1,23 | -2,00 | -1,62 |
| similar to spermine synthase | LOC363469 | -1,23 | -2,00 | -1,62 |
| ribosomal protein s25 | Rps25 | -1,15 | -2,14 | -1,65 |
| FK506 binding protein 3 (predicted) | Fkbp3_predicted | -1,15 | -2,14 | -1,65 |
| similar to prefoldin 4 | LOC364186 | -1,15 | -2,14 | -1,65 |
| Chimerin (chimaerin) 2 | Chn2 | -1,62 | -1,52 | -1,57 |
| glutamyl-prolyl-tRNA synthetase (predicted) | Eprs_predicted | -1,62 | -1,52 | -1,57 |
| N-methylpurine-DNA glycosylase | Mpg | -1,52 | -1,62 | -1,57 |
| apoptosis antagonizing transcription factor | Aatf | -1,52 | -1,62 | -1,57 |
| threonyl-tRNA synthetase | Tars | -1,52 | -1,62 | -1,57 |
| ornithine decarboxylase 1 | Odc1 | -1,32 | -1,74 | -1,53 |
| cyclin G1 | Ccng1 | -1,32 | -1,74 | -1,53 |
| solute carrier family 38, member 2 | Slc38a2 | -1,32 | -1,74 | -1,53 |
| PDZ and LIM domain 1 (elfin) | Pdlim1 | -1,32 | -1,74 | -1,53 |
| farensyl diphosphate synthase | Fdps | -1,32 | -1,74 | -1,53 |
| DnaJ (Hsp40) homolog, subfamily C, member 2 | Dnajc2 | -1,32 | -1,74 | -1,53 |
| lamin B1 | Lmnb1 | -1,32 | -1,74 | -1,53 |
| tumor necrosis factor type 1 receptor associated protein | Trap1 | -1,32 | -1,74 | -1,53 |
| RuvB-like 2 (E. coli) (predicted) | Ruvbl2_predicted | -1,32 | -1,74 | -1,53 |
| WD repeat domain 3 (predicted) | Wdr3_predicted | -1,32 | -1,74 | -1,53 |
| pyrroline-5-carboxylate synthetase (glutamate gamma-semialdehyde synthetase) (predicted) | Pycs_predicted | -1,32 | -1,74 | -1,53 |
