# Supplementary material for: TGF-beta signalling in the adult neurogenic niche promotes stem cell quiescence as well as generation of new neurons
Source: J Cell Mol Med. 2014 Apr 30;18(7):1444–59. doi: 10.1111/jcmm.12298 (PMC4124027; doi:10.1111/jcmm.12298)
Supplement: Supplementary file 9 — Table S3. Hundred most up-regulated genes. [file jcmm0018-1444-SD9.doc]

| **Supp. Table 3. Hundred most up-regulated genes** | |  | | |
| --- | --- | --- | --- | --- |
|  | | Fold change  (TGF-beta1 versus control) | | |
| gene title | gene symbol | experiment 1 | experiment 2 | mean |
| disabled homolog 2 (Drosophila) | dab2 | 7,46 | 12,13 | 9,79 |
| biglycan | Bgn | 9,19 | 6,96 | 8,08 |
| similar to chromosome 20 open reading frame 58 (predicted) | RGD1305809_predicted | 4,59 | 6,96 | 5,78 |
| similar to RIKEN cDNA 1810057C19 | MGC108778 | 4,59 | 5,66 | 5,13 |
| delta-like 1 (Drosophila) | Dll1 | 1,87 | 8,57 | 5,22 |
| N-myc downstream regulated gene 2 | Ndrg2 | 3,73 | 3,73 | 3,73 |
| similar to RIKEN cDNA D330045A20 | LOC315911 | 3,73 | 3,73 | 3,73 |
| similar to hypothetical protein FLJ30973 | LOC363091 | 8,00 | 1,74 | 4,87 |
| Ng23 protein /// similar to Ng23 protein | Ng23 /// LOC499403 | 2,14 | 6,06 | 4,10 |
| crystallin, alpha B | Cryab | 4,92 | 2,64 | 3,78 |
| prostaglandin F2 receptor negative regulator | Ptgfrn | 3,48 | 3,48 | 3,48 |
| similar to SPRY domain-containing SOCS box protein SSB-1 (predicted) | RGD1309319_predicted | 3,48 | 3,25 | 3,37 |
| protein tyrosine phosphatase, non-receptor type 5 | Ptpn5 | 3,03 | 3,48 | 3,26 |
| crystallin, lamda 1 | Cryl1 | 2,00 | 5,28 | 3,64 |
| tenascin R | Tnr | 1,52 | 5,66 | 3,59 |
| calcium/calmodulin-dependent protein kinase I gamma | Camk1g | 2,14 | 4,00 | 3,07 |
| cAMP responsive element binding protein-like 2 (predicted) | Crebl2_predicted | 1,32 | 6,06 | 3,69 |
| quiescin Q6 | Qscn6 | 1,41 | 5,66 | 3,54 |
| Endothelial PAS domain protein 1 | Epas1 | 2,64 | 3,03 | 2,84 |
| Dipeptidylpeptidase 6 | Dpp6 | 2,46 | 3,03 | 2,75 |
| neurexin 1 | Nrxn1 | 1,62 | 4,29 | 2,96 |
| cyclin D2 | Ccnd2 | 2,83 | 2,46 | 2,65 |
| ATPase, Na+/K+ transporting, beta 2 polypeptide | Atp1b2 | 1,62 | 4,00 | 2,81 |
| similar to RIKEN cDNA 6330512M04 gene | LOC293632 | 3,03 | 2,14 | 2,59 |
| septin 8 (predicted) | Sept8_predicted | 2,00 | 3,03 | 2,52 |
| immediate early response 3 | RGD:1303321 | 2,64 | 2,30 | 2,47 |
| Calcium channel, voltage-dependent, gamma subunit 4 | Cacng4 | 1,87 | 3,03 | 2,45 |
| jagged 1 | Jag1 | 2,14 | 2,64 | 2,39 |
| tweety homolog 1 (Drosophila) (predicted) | Ttyh1_predicted | 1,62 | 3,25 | 2,44 |
| cytosolic cysteine dioxygenase 1 | Cdo1 | 2,64 | 2,00 | 2,32 |
| chondroitin sulfate proteoglycan 5 | Cspg5 | 1,52 | 3,25 | 2,38 |
| chondroitin sulfate proteoglycan 2 | Cspg2 | 1,62 | 3,03 | 2,33 |
| procollagen, type IX, alpha 1 (predicted) | Col9a1_predicted | 2,00 | 2,46 | 2,23 |
| Ca<2+-dependent activator protein for secretion | Cadps | 2,14 | 2,30 | 2,22 |
| sodium channel, voltage-gated, type I, beta polypeptide | Scn1b | 2,30 | 2,14 | 2,22 |
| Purkinje cell protein 4 | Pcp4 | 1,87 | 2,46 | 2,16 |
| C1q and tumor necrosis factor related protein 5 (predicted) | C1qtnf5_predicted | 2,00 | 2,30 | 2,15 |
| Calsenilin, presenilin binding protein, EF hand transcription factor | Csen | 2,14 | 2,14 | 2,14 |
| Fas apoptotic inhibitory molecule 2 | Faim2 | 2,14 | 2,14 | 2,14 |
| amyotrophic lateral sclerosis 2 (juvenile) chromosome region, candidate 3 homolog (human) | Als2cr3 | 1,41 | 3,03 | 2,22 |
| ATPase, Na+/K+ transporting, alpha 2 polypeptide | Atp1a2 | 2,00 | 2,14 | 2,07 |
| Syndecan 3 | Sdc3 | 2,00 | 2,14 | 2,07 |
| Similar to rap2 interacting protein x | --- | 1,62 | 2,64 | 2,13 |
| abl-interactor 1 | Abi1 | 1,32 | 3,03 | 2,18 |
| nuclear factor I/X | Nfix | 1,52 | 2,64 | 2,08 |
| Beta-2 microglobulin | B2m | 1,74 | 2,30 | 2,02 |
| protein tyrosine phosphatase, receptor type, N | Ptprn | 2,00 | 2,00 | 2,00 |
| dedicator of cytokinesis 9 | Dock9 | 2,14 | 1,87 | 2,00 |
| Kazal-type serine protease inhibitor domain 1 (predicted) | Kazald1_predicted | 2,14 | 1,87 | 2,00 |
| Similar to RIKEN cDNA 1810054O13 (predicted) | --- | 2,14 | 1,87 | 2,00 |
| synaptosomal-associated protein 25 | Snap25 | 1,74 | 2,14 | 1,94 |
| Monoglyceride lipase | Mgll | 1,74 | 2,14 | 1,94 |
| peptidyl arginine deiminase, type II | Padi2 | 2,14 | 1,74 | 1,94 |
| platelet derived growth factor receptor, alpha polypeptide | Pdgfra | 1,62 | 2,30 | 1,96 |
| low density lipoprotein receptor-related protein 1 (predicted) | Lrp1_predicted | 1,62 | 2,30 | 1,96 |
| ATP-binding cassette, sub-family G (WHITE), member 1 | Abcg1 | 1,87 | 2,00 | 1,93 |
| low density lipoprotein receptor-related protein 4 | Lrp4 | 1,41 | 2,46 | 1,94 |
| syndecan 2 | Sdc2 | 1,62 | 2,14 | 1,88 |
| G0/G1 switch gene 2 (predicted) | G0s2_predicted | 1,74 | 2,00 | 1,87 |
| neurexophilin 1 | Nxph1 | 1,87 | 1,87 | 1,87 |
| Down syndrome cell adhesion molecule | Dscam | 1,87 | 1,87 | 1,87 |
| amiloride-sensitive cation channel 1, neuronal (degenerin) | Accn1 | 1,52 | 2,30 | 1,91 |
| RGD:735085 hypothetical protein | LOC362246 | 1,52 | 2,30 | 1,91 |
| similar to Ras-related protein Rab-27B | LOC363410 | 2,64 | 1,32 | 1,98 |
| low density lipoprotein receptor-related protein 6 (predicted) | Lrp6_predicted | 1,32 | 2,46 | 1,89 |
| glutamate receptor, ionotropic, kainate 4 | Grik4 | 1,87 | 1,74 | 1,80 |
| RAB14, member RAS oncogene family | Rab14 | 1,15 | 2,83 | 1,99 |
| Similar to RIKEN cDNA 5330414D10 (predicted) | --- | 1,15 | 2,83 | 1,99 |
| glutamate receptor, ionotropic, AMPA3 (alpha 3) | Gria3 | 1,23 | 2,64 | 1,94 |
| Bone morphogenetic protein receptor, type 1A | Bmpr1a | 1,23 | 2,64 | 1,94 |
| tyrosine kinase, non-receptor, 2 (predicted) | Tnk2_predicted | 1,41 | 2,30 | 1,86 |
| synaptogyrin 3 (predicted) | Syngr3_predicted | 1,62 | 2,00 | 1,81 |
| glutathione S-transferase, mu 1 | Gstm1 | 1,41 | 2,14 | 1,78 |
| glutamate receptor, ionotropic, 4 | Gria4 | 1,41 | 2,14 | 1,78 |
| tropomodulin 2 | Tmod2 | 1,41 | 2,14 | 1,78 |
| SH3-domain binding protein 4 | Sh3bp4 | 1,41 | 2,14 | 1,78 |
| Fibroblast growth factor receptor 1 | Fgfr1 | 1,41 | 2,14 | 1,78 |
| phospholipase C-like 1 | Plcl1 | 1,62 | 1,87 | 1,75 |
| syntaxin 1B2 | Stx1b2 | 1,74 | 1,74 | 1,74 |
| contactin 6 | Cntn6 | 1,74 | 1,74 | 1,74 |
| transmembrane 4 superfamily member 11 | Tm4sf11 | 1,74 | 1,74 | 1,74 |
| nasal embryonic LHRH factor | Nelf | 1,74 | 1,74 | 1,74 |
| zinc finger and BTB domain containing 4 (predicted) | Zbtb4_predicted | 1,74 | 1,74 | 1,74 |
| similar to maestro | LOC361348 | 1,74 | 1,74 | 1,74 |
| Pyroglutamyl-peptidase I | Pgpep1 | 1,87 | 1,62 | 1,75 |
| diacylglycerol kinase, beta | Dgkb | 2,00 | 1,52 | 1,76 |
| neurotrimin /// hypothetical gene supported by NM_017354 | Hnt /// LOC360435 | 1,23 | 2,30 | 1,76 |
| neuronal growth regulator 1 | Negr1 | 1,23 | 2,30 | 1,76 |
| ER transmembrane protein Dri 42 | Ppap2b | 1,23 | 2,30 | 1,76 |
| potassium voltage gated channel, Shal-related family, member 3 | Kcnd3 | 1,32 | 2,14 | 1,73 |
| TSPY-like 4 (predicted) | Tspyl4_predicted | 1,32 | 2,14 | 1,73 |
| aldolase C, fructose-biphosphate | Aldoc | 1,41 | 2,00 | 1,71 |
| achaete-scute complex homolog-like 1 (Drosophila) | Ascl1 | 1,41 | 2,00 | 1,71 |
| similar to RIKEN cDNA 0610030G03 | LOC287472 | 1,41 | 2,00 | 1,71 |
| leucine rich repeat neuronal 6A (predicted) | Lrrn6a_predicted | 1,41 | 2,00 | 1,71 |
| ATPase, Na+/K+ transporting, beta 1 polypeptide | Atp1b1 | 1,52 | 1,87 | 1,69 |
| erythrocyte protein band 4.1-like 1 | Epb4.1l1 | 1,52 | 1,87 | 1,69 |
| discs, large (Drosophila) homolog-associated protein 1 | Dlgap1 | 1,52 | 1,87 | 1,69 |
| Olf-1/EBF associated Zn finger protein Roaz | RGD:621664 | 1,52 | 1,87 | 1,69 |
| doublecortin | Dcx | 1,23 | 2,14 | 1,69 |
