# Supplementary material for: TGF-beta signalling in the adult neurogenic niche promotes stem cell quiescence as well as generation of new neurons
Source: J Cell Mol Med. 2014 Apr 30;18(7):1444–59. doi: 10.1111/jcmm.12298 (PMC4124027; doi:10.1111/jcmm.12298)
Supplement: Supplementary file 10 — Table S4. Gene Ontology ‘Biological Function’. [file jcmm0018-1444-SD10.doc]

**Supp. Table 4. Gene Ontology “Biological Function“**

| **Term** | **ID** | **Observed** | **Expected** | **ZScore** |
| --- | --- | --- | --- | --- |
| amino acid activation | GO:0043038 | 11 | 0,91 | 10,86 |
| tRNA aminoacylation | GO:0043039 | 11 | 0,91 | 10,86 |
| tRNA aminoacylation for protein translation | GO:0006418 | 11 | 0,91 | 10,86 |
| translation | GO:0043037 | 22 | 3,94 | 9,35 |
| amino acid metabolism | GO:0006520 | 31 | 7,21 | 9,14 |
| tRNA metabolism | GO:0006399 | 12 | 1,63 | 8,32 |
| amino acid and derivative metabolism | GO:0006519 | 36 | 10,42 | 8,2 |
| RNA metabolism | GO:0016070 | 29 | 8,11 | 7,57 |
| biosynthesis | GO:0009058 | 68 | 29,77 | 7,42 |
| nitrogen compound metabolism | GO:0006807 | 38 | 12,69 | 7,38 |
| amine metabolism | GO:0009308 | 36 | 11,74 | 7,34 |
| L-serine metabolism | GO:0006563 | 4 | 0,27 | 7,32 |
| carboxylic acid metabolism | GO:0019752 | 42 | 15,63 | 6,94 |
| organic acid metabolism | GO:0006082 | 42 | 15,73 | 6,9 |
| cellular biosynthesis | GO:0044249 | 59 | 26,65 | 6,61 |
| ribosome biogenesis and assembly | GO:0042254 | 8 | 1,18 | 6,44 |
| cholesterol biosynthesis | GO:0006695 | 7 | 0,95 | 6,35 |
| sterol biosynthesis | GO:0016126 | 7 | 1 | 6,16 |
| base-excision repair | GO:0006284 | 6 | 0,77 | 6,1 |
| amino acid biosynthesis | GO:0008652 | 7 | 1,09 | 5,81 |
| purine nucleotide biosynthesis | GO:0006164 | 7 | 1,13 | 5,65 |
| ribonucleoside monophosphate biosynthesis | GO:0009156 | 4 | 0,45 | 5,39 |
| protein biosynthesis | GO:0006412 | 33 | 14,09 | 5,24 |
| purine nucleotide metabolism | GO:0006163 | 8 | 1,59 | 5,22 |
| serine family amino acid metabolism | GO:0009069 | 5 | 0,73 | 5,14 |
| aspartate family amino acid metabolism | GO:0009066 | 4 | 0,5 | 5,08 |
| nucleoside monophosphate biosynthesis | GO:0009124 | 4 | 0,5 | 5,08 |
| ribonucleoside monophosphate metabolism | GO:0009161 | 4 | 0,5 | 5,08 |
| nucleocytoplasmic transport | GO:0006913 | 13 | 3,63 | 5,06 |
| nuclear transport | GO:0051169 | 12 | 3,26 | 4,97 |
| protein folding | GO:0006457 | 13 | 3,85 | 4,79 |
| RNA processing | GO:0006396 | 18 | 6,39 | 4,73 |
| nucleobase, nucleoside, nucleotide and nucleic acid metabolism | GO:0006139 | 98 | 64,22 | 4,66 |
| macromolecule biosynthesis | GO:0009059 | 34 | 16,18 | 4,62 |
| nucleoside monophosphate metabolism | GO:0009123 | 4 | 0,59 | 4,55 |
| nitrogen compound biosynthesis | GO:0044271 | 9 | 2,36 | 4,44 |
| amine biosynthesis | GO:0009309 | 9 | 2,36 | 4,44 |
| protein import | GO:0017038 | 9 | 2,36 | 4,44 |
| transport | GO:0006810 | 96 | 64,08 | 4,4 |
| nucleotide biosynthesis | GO:0009165 | 10 | 2,81 | 4,4 |
| ribosome biogenesis | GO:0007046 | 5 | 0,91 | 4,4 |
| steroid biosynthesis | GO:0006694 | 9 | 2,49 | 4,23 |
| rRNA processing | GO:0006364 | 4 | 0,68 | 4,12 |
| cholesterol metabolism | GO:0008203 | 9 | 2,58 | 4,1 |
| translational initiation | GO:0006413 | 6 | 1,36 | 4,08 |
| protein import into nucleus | GO:0006606 | 8 | 2,18 | 4,05 |
| nuclear import | GO:0051170 | 8 | 2,18 | 4,05 |
| cytoplasm organization and biogenesis | GO:0007028 | 8 | 2,22 | 3,98 |
| sterol metabolism | GO:0016125 | 9 | 2,72 | 3,91 |
| establishment of cellular localization | GO:0051649 | 32 | 16,77 | 3,88 |
| cellular localization | GO:0051641 | 32 | 16,95 | 3,81 |
| cell growth | GO:0016049 | 13 | 4,89 | 3,77 |
| biopolymer metabolism | GO:0043283 | 78 | 52,84 | 3,77 |
| rRNA metabolism | GO:0016072 | 4 | 0,77 | 3,77 |
| protein import into nucleus, translocation | GO:0000060 | 4 | 0,77 | 3,77 |
| alcohol metabolism | GO:0006066 | 19 | 8,43 | 3,76 |
| cell organization and biogenesis | GO:0016043 | 73 | 48,94 | 3,73 |
| regulation of cell size | GO:0008361 | 13 | 5,03 | 3,66 |
| growth | GO:0040007 | 18 | 8,02 | 3,64 |
| cell proliferation | GO:0008283 | 41 | 24,02 | 3,64 |
| amino acid catabolism | GO:0009063 | 6 | 1,59 | 3,59 |
| mitotic cell cycle | GO:0000278 | 16 | 6,89 | 3,58 |
| cellular protein metabolism | GO:0044267 | 81 | 56,69 | 3,53 |
| cell cycle | GO:0007049 | 34 | 19,31 | 3,5 |
| chromosome segregation | GO:0007059 | 5 | 1,22 | 3,5 |
| steroid hormone receptor signaling pathway | GO:0030518 | 5 | 1,22 | 3,5 |
| glucose catabolism | GO:0006007 | 5 | 1,22 | 3,5 |
| regulation of progression through cell cycle | GO:0000074 | 24 | 12,24 | 3,49 |
| protein metabolism | GO:0019538 | 88 | 62,95 | 3,48 |
| regulation of cell cycle | GO:0051726 | 24 | 12,28 | 3,47 |
| protein targeting | GO:0006605 | 14 | 5,85 | 3,47 |
| glycolysis | GO:0006096 | 4 | 0,86 | 3,47 |
| regulation of translational initiation | GO:0006446 | 4 | 0,86 | 3,47 |
| amine catabolism | GO:0009310 | 7 | 2,18 | 3,36 |
| cellular catabolism | GO:0044248 | 24 | 12,6 | 3,33 |
| cellular macromolecule metabolism | GO:0044260 | 81 | 57,92 | 3,32 |
| nucleotide metabolism | GO:0009117 | 12 | 4,89 | 3,3 |
| intracellular transport | GO:0046907 | 29 | 16,36 | 3,26 |
| nitrogen compound catabolism | GO:0044270 | 7 | 2,27 | 3,23 |
| cell division | GO:0051301 | 9 | 3,31 | 3,21 |
| intracellular receptor-mediated signaling pathway | GO:0030522 | 5 | 1,4 | 3,11 |
| monosaccharide catabolism | GO:0046365 | 5 | 1,4 | 3,11 |
| hexose catabolism | GO:0019320 | 5 | 1,4 | 3,11 |
| DNA replication | GO:0006260 | 10 | 3,99 | 3,09 |
| DNA damage response, signal transduction | GO:0042770 | 4 | 1 | 3,08 |
| glutamine family amino acid metabolism | GO:0009064 | 4 | 1 | 3,08 |
| DNA metabolism | GO:0006259 | 25 | 13,96 | 3,07 |
| lipid transport | GO:0006869 | 6 | 1,9 | 3,05 |
| amine transport | GO:0015837 | 7 | 2,4 | 3,04 |
| catabolism | GO:0009056 | 26 | 14,82 | 3,02 |
| alcohol catabolism | GO:0046164 | 5 | 1,45 | 3,02 |
| nervous system development | GO:0007399 | 42 | 27,15 | 3,01 |
| mitosis | GO:0007067 | 9 | 3,53 | 2,99 |
| organelle organization and biogenesis | GO:0006996 | 35 | 21,75 | 2,98 |
| glutamate signaling pathway | GO:0007215 | 4 | 1,04 | 2,97 |
| nuclear export | GO:0051168 | 4 | 1,04 | 2,97 |
| M phase of mitotic cell cycle | GO:0000087 | 9 | 3,58 | 2,94 |
| system development | GO:0048731 | 43 | 28,41 | 2,89 |
| negative regulation of enzyme activity | GO:0043086 | 7 | 2,54 | 2,87 |
| Notch signaling pathway | GO:0007219 | 5 | 1,54 | 2,86 |
| ribonucleotide metabolism | GO:0009259 | 5 | 1,54 | 2,86 |
| regulation of cell proliferation | GO:0042127 | 26 | 15,45 | 2,79 |
| nuclear mRNA splicing, via spliceosome | GO:0000398 | 6 | 2,08 | 2,78 |
| RNA splicing, via transesterification reactions | GO:0000375 | 6 | 2,08 | 2,78 |
| RNA splicing, via transesterification reactions with bulged adenosine as nucleophile | GO:0000377 | 6 | 2,08 | 2,78 |
| response to oxidative stress | GO:0006979 | 7 | 2,63 | 2,77 |
| macromolecule catabolism | GO:0009057 | 15 | 7,66 | 2,74 |
| anterior/posterior pattern formation | GO:0009952 | 6 | 2,13 | 2,72 |
| interphase | GO:0051325 | 7 | 2,72 | 2,66 |
| interphase of mitotic cell cycle | GO:0051329 | 7 | 2,72 | 2,66 |
| apoptotic program | GO:0008632 | 6 | 2,18 | 2,66 |
| lipid biosynthesis | GO:0008610 | 13 | 6,57 | 2,59 |
| intracellular protein transport | GO:0006886 | 18 | 10,06 | 2,59 |
| ribonucleotide biosynthesis | GO:0009260 | 4 | 1,22 | 2,57 |
| purine ribonucleotide metabolism | GO:0009150 | 4 | 1,22 | 2,57 |
| amino acid transport | GO:0006865 | 5 | 1,72 | 2,56 |
| cell death | GO:0008219 | 34 | 22,48 | 2,55 |
| programmed cell death | GO:0012501 | 33 | 21,71 | 2,54 |
| death | GO:0016265 | 34 | 22,8 | 2,46 |
| response to endogenous stimulus | GO:0009719 | 14 | 7,48 | 2,46 |
| steroid metabolism | GO:0008202 | 11 | 5,44 | 2,46 |
| mRNA processing | GO:0006397 | 8 | 3,53 | 2,44 |
| regulated secretory pathway | GO:0045055 | 7 | 2,95 | 2,43 |
| apoptosis | GO:0006915 | 32 | 21,34 | 2,42 |
| central nervous system development | GO:0007417 | 14 | 7,57 | 2,41 |
| M phase | GO:0000279 | 11 | 5,53 | 2,4 |
| response to DNA damage stimulus | GO:0006974 | 13 | 6,93 | 2,38 |
| regulation of muscle contraction | GO:0006937 | 4 | 1,36 | 2,32 |
| regulation of transport | GO:0051049 | 8 | 3,67 | 2,32 |
| regulation of translation | GO:0006445 | 5 | 1,9 | 2,3 |
| RNA splicing | GO:0008380 | 7 | 3,08 | 2,29 |
| regulation of cell growth | GO:0001558 | 8 | 3,76 | 2,25 |
| neurotransmitter transport | GO:0006836 | 5 | 1,95 | 2,24 |
| glucose metabolism | GO:0006006 | 7 | 3,17 | 2,21 |
| MAPKKK cascade | GO:0000165 | 10 | 5,21 | 2,16 |
| negative regulation of apoptosis | GO:0043066 | 12 | 6,66 | 2,13 |
| response to hypoxia | GO:0001666 | 4 | 1,5 | 2,1 |
| mRNA metabolism | GO:0016071 | 8 | 3,94 | 2,1 |
| potassium ion transport | GO:0006813 | 8 | 3,94 | 2,1 |
| negative regulation of programmed cell death | GO:0043069 | 12 | 6,75 | 2,08 |
| organic acid transport | GO:0015849 | 6 | 2,72 | 2,04 |
| carboxylic acid transport | GO:0046942 | 6 | 2,72 | 2,04 |
| main pathways of carbohydrate metabolism | GO:0006092 | 6 | 2,72 | 2,04 |
| protein transport | GO:0015031 | 20 | 12,96 | 2,03 |
| DNA-dependent DNA replication | GO:0006261 | 5 | 2,13 | 2,02 |
| cellular lipid metabolism | GO:0044255 | 23 | 15,45 | 2 |
| lipid metabolism | GO:0006629 | 27 | 18,72 | 2 |
| regulation of growth | GO:0040008 | 10 | 5,48 | 1,99 |
| oxygen and reactive oxygen species metabolism | GO:0006800 | 7 | 3,49 | 1,93 |
| cellular carbohydrate catabolism | GO:0044275 | 5 | 2,22 | 1,91 |
| regulation of caspase activity | GO:0043281 | 4 | 1,63 | 1,9 |
| cellular macromolecule catabolism | GO:0044265 | 11 | 6,34 | 1,9 |
| metal ion transport | GO:0030001 | 17 | 10,97 | 1,89 |
| carbohydrate catabolism | GO:0016052 | 5 | 2,27 | 1,86 |
| monosaccharide metabolism | GO:0005996 | 8 | 4,26 | 1,86 |
| hexose metabolism | GO:0019318 | 8 | 4,26 | 1,86 |
| protein oligomerization | GO:0051259 | 4 | 1,68 | 1,84 |
| biopolymer catabolism | GO:0043285 | 10 | 5,76 | 1,82 |
| chromosome organization and biogenesis | GO:0051276 | 10 | 5,76 | 1,82 |
| regulation of enzyme activity | GO:0050790 | 15 | 9,61 | 1,8 |
| regulation of adenylate cyclase activity | GO:0045761 | 4 | 1,72 | 1,78 |
| neuron maturation | GO:0042551 | 4 | 1,72 | 1,78 |
| negative regulation of biological process | GO:0048519 | 43 | 33,4 | 1,77 |
| response to stress | GO:0006950 | 47 | 37,02 | 1,75 |
| development | GO:0007275 | 96 | 82,16 | 1,73 |
| transmission of nerve impulse | GO:0019226 | 21 | 14,68 | 1,72 |
| regulation of cyclase activity | GO:0031279 | 4 | 1,81 | 1,67 |
| regulation of lyase activity | GO:0051339 | 4 | 1,81 | 1,67 |
| establishment of protein localization | GO:0045184 | 20 | 14 | 1,67 |
| intracellular signaling cascade | GO:0007242 | 41 | 32,09 | 1,67 |
| vesicle-mediated transport | GO:0016192 | 20 | 14,09 | 1,64 |
| endocytosis | GO:0006897 | 9 | 5,39 | 1,6 |
| cell maturation | GO:0048469 | 6 | 3,22 | 1,59 |
| embryonic development (sensu Metazoa) | GO:0009792 | 8 | 4,71 | 1,56 |
| protein localization | GO:0008104 | 21 | 15,18 | 1,55 |
| regulation of programmed cell death | GO:0043067 | 23 | 16,9 | 1,55 |
| regulation of biosynthesis | GO:0009889 | 9 | 5,53 | 1,52 |
| DNA repair | GO:0006281 | 9 | 5,53 | 1,52 |
| protein complex assembly | GO:0006461 | 11 | 7,07 | 1,52 |
| synaptic transmission | GO:0007268 | 19 | 13,64 | 1,51 |
| neurotransmitter secretion | GO:0007269 | 5 | 2,63 | 1,5 |
| DNA recombination | GO:0006310 | 4 | 1,99 | 1,46 |
| negative regulation of cellular process | GO:0048523 | 37 | 29,77 | 1,4 |
| regulation of apoptosis | GO:0042981 | 22 | 16,54 | 1,4 |
| regulation of protein metabolism | GO:0051246 | 12 | 8,16 | 1,39 |
| regulation of cellular biosynthesis | GO:0031326 | 8 | 4,98 | 1,39 |
| neurogenesis | GO:0048699 | 18 | 13,19 | 1,38 |
| response to chemical stimulus | GO:0042221 | 19 | 14,09 | 1,36 |
| anti-apoptosis | GO:0006916 | 8 | 5,12 | 1,31 |
| fatty acid metabolism | GO:0006631 | 9 | 5,94 | 1,3 |
| negative regulation of cell proliferation | GO:0008285 | 10 | 6,75 | 1,29 |
| regulation of hydrolase activity | GO:0051336 | 4 | 2,22 | 1,23 |
| protein kinase cascade | GO:0007243 | 15 | 11,06 | 1,23 |
| amino acid derivative metabolism | GO:0006575 | 6 | 3,72 | 1,22 |
| positive regulation of development | GO:0051094 | 5 | 2,99 | 1,19 |
| regulation of protein biosynthesis | GO:0006417 | 7 | 4,53 | 1,19 |
| regulation of MAPK activity | GO:0043405 | 5 | 2,99 | 1,19 |
| response to abiotic stimulus | GO:0009628 | 23 | 18,17 | 1,18 |
| coenzyme metabolism | GO:0006732 | 6 | 3,81 | 1,16 |
| energy derivation by oxidation of organic compounds | GO:0015980 | 7 | 4,62 | 1,14 |
| cell differentiation | GO:0030154 | 40 | 33,81 | 1,13 |
| cellular morphogenesis | GO:0000902 | 20 | 15,73 | 1,12 |
| di-, tri-valent inorganic cation transport | GO:0015674 | 7 | 4,71 | 1,08 |
| secretory pathway | GO:0045045 | 11 | 8,07 | 1,07 |
| negative regulation of protein metabolism | GO:0051248 | 4 | 2,4 | 1,06 |
| negative regulation of signal transduction | GO:0009968 | 5 | 3,17 | 1,05 |
| cell communication | GO:0007154 | 99 | 90,36 | 1,04 |
| neuron differentiation | GO:0030182 | 15 | 11,65 | 1,02 |
| establishment and/or maintenance of chromatin architecture | GO:0006325 | 6 | 4,08 | 0,98 |
| cell fate commitment | GO:0045165 | 5 | 3,31 | 0,96 |
| tube morphogenesis | GO:0035239 | 4 | 2,54 | 0,94 |
| cell motility | GO:0006928 | 17 | 13,69 | 0,93 |
| localization of cell | GO:0051674 | 17 | 13,69 | 0,93 |
| cation transport | GO:0006812 | 17 | 13,69 | 0,93 |
| locomotion | GO:0040011 | 17 | 13,87 | 0,87 |
| extracellular structure organization and biogenesis | GO:0043062 | 4 | 2,63 | 0,87 |
| extracellular matrix organization and biogenesis | GO:0030198 | 4 | 2,63 | 0,87 |
| learning and/or memory | GO:0007611 | 4 | 2,63 | 0,87 |
| negative regulation of cellular physiological process | GO:0051243 | 30 | 25,79 | 0,87 |
| morphogenesis | GO:0009653 | 42 | 37,12 | 0,86 |
| DNA packaging | GO:0006323 | 6 | 4,31 | 0,84 |
| regulation of neurotransmitter levels | GO:0001505 | 6 | 4,31 | 0,84 |
| neuron development | GO:0048666 | 11 | 8,66 | 0,82 |
| protein catabolism | GO:0030163 | 6 | 4,35 | 0,81 |
| embryonic development (sensu Vertebrata) | GO:0043009 | 4 | 2,72 | 0,8 |
| regulation of signal transduction | GO:0009966 | 11 | 8,75 | 0,79 |
| regulation of protein kinase activity | GO:0045859 | 7 | 5,26 | 0,78 |
| exocytosis | GO:0006887 | 5 | 3,58 | 0,77 |
| pattern specification | GO:0007389 | 7 | 5,3 | 0,76 |
| chromosome organization and biogenesis (sensu Eukaryota) | GO:0007001 | 7 | 5,3 | 0,76 |
| negative regulation of physiological process | GO:0043118 | 31 | 27,28 | 0,75 |
| generation of precursor metabolites and energy | GO:0006091 | 14 | 11,6 | 0,73 |
| regulation of kinase activity | GO:0043549 | 7 | 5,39 | 0,71 |
| positive regulation of signal transduction | GO:0009967 | 4 | 2,86 | 0,7 |
| signal transduction | GO:0007165 | 83 | 77,63 | 0,69 |
| sexual reproduction | GO:0019953 | 11 | 9,02 | 0,68 |
| regulation of transferase activity | GO:0051338 | 7 | 5,48 | 0,67 |
| proteolysis | GO:0006508 | 15 | 12,73 | 0,66 |
| cell development | GO:0048468 | 16 | 13,69 | 0,65 |
| monovalent inorganic cation transport | GO:0015672 | 10 | 8,2 | 0,65 |
| axonogenesis | GO:0007409 | 7 | 5,62 | 0,6 |
| ubiquitin cycle | GO:0006512 | 5 | 3,85 | 0,6 |
| cell adhesion | GO:0007155 | 19 | 16,77 | 0,57 |
| transcription from RNA polymerase II promoter | GO:0006366 | 22 | 19,67 | 0,55 |
| cofactor metabolism | GO:0051186 | 6 | 4,85 | 0,54 |
| gametogenesis | GO:0007276 | 9 | 7,61 | 0,52 |
| G-protein signaling, coupled to cAMP nucleotide second messenger | GO:0007188 | 5 | 3,99 | 0,52 |
| cellular carbohydrate metabolism | GO:0044262 | 10 | 8,57 | 0,51 |
| neuron morphogenesis during differentiation | GO:0048667 | 8 | 6,75 | 0,5 |
| neurite morphogenesis | GO:0031175 | 8 | 6,75 | 0,5 |
| morphogenesis of an epithelium | GO:0002009 | 4 | 3,17 | 0,48 |
| second-messenger-mediated signaling | GO:0019932 | 11 | 9,56 | 0,48 |
| ion transport | GO:0006811 | 21 | 19,08 | 0,46 |
| embryonic development | GO:0009790 | 12 | 10,7 | 0,41 |
| transmembrane receptor protein tyrosine kinase signaling pathway | GO:0007169 | 7 | 6,03 | 0,41 |
| tube development | GO:0035295 | 5 | 4,21 | 0,39 |
| sulfur metabolism | GO:0006790 | 4 | 3,31 | 0,39 |
| electron transport | GO:0006118 | 6 | 5,17 | 0,38 |
| G-protein signaling, coupled to cyclic nucleotide second messenger | GO:0007187 | 6 | 5,21 | 0,36 |
| secretion | GO:0046903 | 12 | 10,88 | 0,35 |
| carbohydrate metabolism | GO:0005975 | 12 | 10,92 | 0,34 |
| peptidyl-amino acid modification | GO:0018193 | 4 | 3,4 | 0,33 |
| calcium ion transport | GO:0006816 | 4 | 3,4 | 0,33 |
| enzyme linked receptor protein signaling pathway | GO:0007167 | 10 | 9,06 | 0,32 |
| brain development | GO:0007420 | 5 | 4,4 | 0,3 |
| negative regulation of progression through cell cycle | GO:0045786 | 4 | 3,49 | 0,28 |
| proteolysis during cellular protein catabolism | GO:0051603 | 4 | 3,49 | 0,28 |
| response to stimulus | GO:0050896 | 69 | 67,16 | 0,25 |
| blood vessel development | GO:0001568 | 6 | 5,44 | 0,25 |
| cellular protein catabolism | GO:0044257 | 4 | 3,53 | 0,25 |
| cAMP-mediated signaling | GO:0019933 | 5 | 4,49 | 0,25 |
| positive regulation of biological process | GO:0048518 | 35 | 33,67 | 0,24 |
| embryonic morphogenesis | GO:0048598 | 5 | 4,58 | 0,2 |
| regulation of transcription from RNA polymerase II promoter | GO:0006357 | 16 | 15,27 | 0,19 |
| vasculature development | GO:0001944 | 6 | 5,62 | 0,17 |
| regulation of metabolism | GO:0019222 | 48 | 46,95 | 0,17 |
| cellular morphogenesis during differentiation | GO:0000904 | 8 | 7,57 | 0,16 |
| male gamete generation | GO:0048232 | 6 | 5,66 | 0,15 |
| spermatogenesis | GO:0007283 | 6 | 5,66 | 0,15 |
| cell migration | GO:0016477 | 10 | 9,61 | 0,13 |
| positive regulation of enzyme activity | GO:0043085 | 6 | 5,71 | 0,12 |
| cell-cell signaling | GO:0007267 | 25 | 24,47 | 0,11 |
| cyclic-nucleotide-mediated signaling | GO:0019935 | 6 | 5,76 | 0,11 |
| regulation of organismal physiological process | GO:0051239 | 12 | 11,74 | 0,08 |
| positive regulation of cell proliferation | GO:0008284 | 7 | 6,8 | 0,08 |
| negative regulation of metabolism | GO:0009892 | 11 | 10,83 | 0,05 |
| positive regulation of cellular process | GO:0048522 | 29 | 28,78 | 0,04 |
| regulation of development | GO:0050793 | 9 | 8,93 | 0,03 |
| protein amino acid phosphorylation | GO:0006468 | 14 | 13,91 | 0,02 |
| membrane lipid metabolism | GO:0006643 | 4 | 3,99 | 0,01 |
| induction of programmed cell death | GO:0012502 | 7 | 6,98 | 0,01 |
| induction of apoptosis | GO:0006917 | 7 | 6,98 | 0,01 |
| muscle contraction | GO:0006936 | 5 | 5,03 | -0,01 |
| phosphorylation | GO:0016310 | 16 | 16,04 | -0,01 |
| neurophysiological process | GO:0050877 | 25 | 25,11 | -0,02 |
| reproduction | GO:0000003 | 12 | 12,28 | -0,08 |
| positive regulation of apoptosis | GO:0043065 | 8 | 8,38 | -0,14 |
| cell surface receptor linked signal transduction | GO:0007166 | 39 | 39,97 | -0,16 |
| regulation of cellular metabolism | GO:0031323 | 43 | 44,14 | -0,18 |
| positive regulation of programmed cell death | GO:0043068 | 8 | 8,52 | -0,18 |
| tissue remodeling | GO:0048771 | 4 | 4,4 | -0,19 |
| negative regulation of transcription from RNA polymerase II promoter | GO:0000122 | 4 | 4,4 | -0,19 |
| behavior | GO:0007610 | 11 | 11,69 | -0,21 |
| wound healing | GO:0042060 | 4 | 4,58 | -0,28 |
| cytoskeleton organization and biogenesis | GO:0007010 | 10 | 11,06 | -0,33 |
| blood vessel morphogenesis | GO:0048514 | 4 | 4,71 | -0,34 |
| cell homeostasis | GO:0019725 | 6 | 6,93 | -0,37 |
| phosphorus metabolism | GO:0006793 | 18 | 19,76 | -0,41 |
| phosphate metabolism | GO:0006796 | 18 | 19,76 | -0,41 |
| regulation of cell differentiation | GO:0045595 | 6 | 7,11 | -0,43 |
| positive regulation of cellular physiological process | GO:0051242 | 24 | 26,1 | -0,43 |
| transcription | GO:0006350 | 39 | 41,6 | -0,43 |
| positive regulation of physiological process | GO:0043119 | 25 | 27,46 | -0,5 |
| negative regulation of cellular metabolism | GO:0031324 | 8 | 9,52 | -0,51 |
| hemopoiesis | GO:0030097 | 5 | 6,39 | -0,57 |
| tissue development | GO:0009888 | 9 | 10,83 | -0,58 |
| positive regulation of transcription from RNA polymerase II promoter | GO:0045944 | 4 | 5,3 | -0,58 |
| transcription, DNA-dependent | GO:0006351 | 33 | 36,35 | -0,59 |
| homeostasis | GO:0042592 | 8 | 9,88 | -0,62 |
| circulation | GO:0008015 | 4 | 5,66 | -0,72 |
| response to external stimulus | GO:0009605 | 17 | 20,12 | -0,73 |
| regulation of nucleobase, nucleoside, nucleotide and nucleic acid metabolism | GO:0019219 | 34 | 38,2 | -0,73 |
| regulation of transcription | GO:0045449 | 33 | 37,21 | -0,74 |
| cation homeostasis | GO:0030003 | 4 | 5,76 | -0,75 |
| negative regulation of transcription, DNA-dependent | GO:0045892 | 4 | 5,76 | -0,75 |
| response to wounding | GO:0009611 | 13 | 15,95 | -0,77 |
| negative regulation of nucleobase, nucleoside, nucleotide and nucleic acid metabolism | GO:0045934 | 6 | 8,11 | -0,77 |
| hemopoietic or lymphoid organ development | GO:0048534 | 5 | 7,02 | -0,79 |
| small GTPase mediated signal transduction | GO:0007264 | 4 | 5,85 | -0,79 |
| positive regulation of transcription | GO:0045941 | 7 | 9,43 | -0,82 |
| cell ion homeostasis | GO:0006873 | 4 | 6,03 | -0,85 |
| positive regulation of nucleobase, nucleoside, nucleotide and nucleic acid metabolism | GO:0045935 | 7 | 9,61 | -0,87 |
| regulation of transcription, DNA-dependent | GO:0006355 | 28 | 32,95 | -0,92 |
| positive regulation of transcription, DNA-dependent | GO:0045893 | 5 | 7,61 | -0,98 |
| biopolymer modification | GO:0043412 | 26 | 31,22 | -0,99 |
| ion homeostasis | GO:0050801 | 4 | 6,53 | -1,02 |
| immune cell activation | GO:0045321 | 4 | 6,53 | -1,02 |
| organ morphogenesis | GO:0009887 | 15 | 19,4 | -1,04 |
| cell activation | GO:0001775 | 4 | 6,62 | -1,05 |
| negative regulation of transcription | GO:0016481 | 5 | 7,84 | -1,05 |
| positive regulation of cellular metabolism | GO:0031325 | 8 | 11,74 | -1,13 |
| protein modification | GO:0006464 | 24 | 29,91 | -1,14 |
| skeletal development | GO:0001501 | 5 | 8,2 | -1,15 |
| inflammatory response | GO:0006954 | 5 | 8,43 | -1,22 |
| positive regulation of metabolism | GO:0009893 | 8 | 12,28 | -1,27 |
| G-protein coupled receptor protein signaling pathway | GO:0007186 | 14 | 19,71 | -1,35 |
| sensory perception | GO:0007600 | 5 | 10,97 | -1,87 |
| organ development | GO:0048513 | 28 | 39,34 | -1,94 |
| response to pest, pathogen or parasite | GO:0009613 | 8 | 16,63 | -2,21 |
| response to other organism | GO:0051707 | 8 | 17,27 | -2,33 |
| defense response | GO:0006952 | 13 | 26,15 | -2,71 |
| response to biotic stimulus | GO:0009607 | 14 | 27,51 | -2,72 |
| immune response | GO:0006955 | 12 | 24,88 | -2,72 |
| organismal physiological process | GO:0050874 | 48 | 68,34 | -2,73 |
