# Supplementary material for: TGF-beta signalling in the adult neurogenic niche promotes stem cell quiescence as well as generation of new neurons
Source: J Cell Mol Med. 2014 Apr 30;18(7):1444–59. doi: 10.1111/jcmm.12298 (PMC4124027; doi:10.1111/jcmm.12298)
Supplement: Supplementary file 12 — Table S6. TGF-β1 regulated genes ‘cell growth’. [file jcmm0018-1444-SD12.doc]

| **Supp. Table 6.**  **TGF-beta1 regulated genes “cell growth”** | |
| --- | --- |
| **cell growth: z=3.01; p=0.003; fdr=0,008** | |
| gene title | regulation |
| chondroitin sulfate proteoglycan 5 | **↑** |
| cyclin E | **↓** |
| cyclin G1 | **↓** |
| Disabled homolog 2 (Drosophila) | **↑** |
| DnaJ (Hsp40) homolog, subfamily C, member 2 | **↓** |
| Fibroblast growth factor receptor 1 | **↑** |
| nucleolar protein 5 | **↓** |
| RuvB-like protein 1 | **↓** |
| solute carrier family 3 (activators of dibasic and neutral amino acid transport), member 2 | **↓** |
| suppressor of cytokine signaling 2 | **↓** |
| tumor protein p53 | **↓** |
| v-akt murine thymoma viral oncogene homolog 1 | **↑** |
