# Supplementary material for: TGF-beta signalling in the adult neurogenic niche promotes stem cell quiescence as well as generation of new neurons
Source: J Cell Mol Med. 2014 Apr 30;18(7):1444–59. doi: 10.1111/jcmm.12298 (PMC4124027; doi:10.1111/jcmm.12298)
Supplement: Supplementary file 13 — Table S7. TGF-β1 regulated genes ‘cell cycle’. [file jcmm0018-1444-SD13.doc]

| **Supp. Table 7.**  **TGF-beta1 regulated genes “cell cycle”** | |
| --- | --- |
| **regulation of cell cycle: z=1.98; p=0.048; fdr=0,084** | |
| gene title | regulation |
| apoptosis antagonizing transcription factor | **↓** |
| cell division cycle 20 homolog (S. cerevisiae) | **↓** |
| chaperonin containing TCP1, subunit 2 (beta) | **↓** |
| cyclin B1 | **↓** |
| cyclin D2 | **↑** |
| cyclin E | **↓** |
| cyclin G1 | **↓** |
| cyclin-dependent kinase inhibitor 1C (P57) | **↓** |
| guanine nucleotide binding protein-like 3 (nucleolar) | **↓** |
| Hexokinase 2 | **↓** |
| Inhibitor of DNA binding 3, dominant negative helix-loop-helix protein | **↓** |
| mitogen activated protein kinase 3 | **↓** |
| myelocytomatosis viral oncogene homolog (avian) | **↓** |
| nuclear autoantigenic sperm protein | **↓** |
| polo-like kinase 1 (Drosophila) | **↓** |
| prothymosin alpha | **↓** |
| quiescin Q6 | **↑** |
| signal transducer and activator of transcription 1 | **↑** |
| tumor protein p53 | **↓** |
| von Hippel-Lindau syndrome homolog | **↑** |
