# Supplementary material for: TGF-beta signalling in the adult neurogenic niche promotes stem cell quiescence as well as generation of new neurons
Source: J Cell Mol Med. 2014 Apr 30;18(7):1444–59. doi: 10.1111/jcmm.12298 (PMC4124027; doi:10.1111/jcmm.12298)
Supplement: Supplementary file 14 — Table S8. TGF-β1 regulated genes ‘regulation of cell proliferation’. [file jcmm0018-1444-SD14.doc]

| **Supp. Table 8.**  **TGF-beta1 regulated genes “regulation of cell proliferation”** | |
| --- | --- |
| **regulation of cell proliferation: z=1.72; p=0.085; fdr=0,125** | |
| gene title | regulation |
| 7-dehydrocholesterol reductase | **↓** |
| acidic nuclear phosphoprotein 32 family, member B | **↓** |
| caveolin | **↑** |
| cellular nucleic acid binding protein 1 | **↓** |
| cyclin-dependent kinase inhibitor 1C (P57) | **↓** |
| Discs, large homolog 3 (Drosophila) | **↑** |
| endothelial differentiation, sphingolipid G-protein-coupled receptor, 5 | **↑** |
| Fibroblast growth factor receptor 1 | **↑** |
| fibroblast growth factor receptor-like 1 | **↑** |
| guanine nucleotide binding protein-like 3 (nucleolar) | **↓** |
| hairy and enhancer of split 1 (Drosophila) | **↑** |
| immediate early response 3 | **↑** |
| Inhibitor of DNA binding 3, dominant negative helix-loop-helix protein | **↓** |
| jagged 1 | **↑** |
| macrophage migration inhibitory factor | **↓** |
| myelocytomatosis viral oncogene homolog (avian) | **↓** |
| nucleophosmin 1 /// similar to Nucleophosmin (NPM) (Nucleolar phosphoprotein B23) (Numatrin) (Nucleolar protein NO38) | **↓** |
| nucleosome assembly protein 1-like 1 | **↓** |
| phosphatidic acid phosphatase 2a | **↓** |
| quiescin Q6 | **↑** |
| TGFB inducible early growth response | **↑** |
| Transforming growth factor, beta receptor II | **↓** |
| tumor protein p53 | **↓** |
| v-myc myelocytomatosis viral related oncogene, neuroblastoma derived (avian) | **↑** |
