# Supplementary material for: TGF-beta signalling in the adult neurogenic niche promotes stem cell quiescence as well as generation of new neurons
Source: J Cell Mol Med. 2014 Apr 30;18(7):1444–59. doi: 10.1111/jcmm.12298 (PMC4124027; doi:10.1111/jcmm.12298)
Supplement: Supplementary file 15 — Table S9. TGF-β1 regulated genes ‘neuron maturation’. [file jcmm0018-1444-SD15.doc]

| **Supp. Table 9.**  **TGF-beta1 regulated genes “neuron maturation”** | |
| --- | --- |
| **neuron maturation: z=2.46; p=0.014; fdr=0,033** | |
| gene title | regulation |
| agrin | **↑** |
| CD9 antigen | **↑** |
| cyclin-dependent kinase inhibitor 1C (P57) | **↓** |
| myelin basic protein | **↓** |
| neuroligin 1 | **↑** |
| transmembrane 4 superfamily member 11 | **↑** |
