# Supplementary material for: TGF-beta signalling in the adult neurogenic niche promotes stem cell quiescence as well as generation of new neurons
Source: J Cell Mol Med. 2014 Apr 30;18(7):1444–59. doi: 10.1111/jcmm.12298 (PMC4124027; doi:10.1111/jcmm.12298)
Supplement: Supplementary file 16 — Table S10. TGF-β1 regulated genes ‘neuron differentiation’. [file jcmm0018-1444-SD16.doc]

| **Supp. Table 10.**  **TGF-beta1 regulated genes “neuron differentiation”** | |
| --- | --- |
| **neuron differentiation: z=1.45; p=0.147; fdr=0,185** | |
| gene title | regulation |
| achaete-scute complex homolog-like 1 (Drosophila) | **↑** |
| agrin | **↑** |
| CD9 antigen | **↑** |
| chondroitin sulfate proteoglycan 5 | **↑** |
| cyclin-dependent kinase inhibitor 1C (P57) | **↓** |
| Dihydropyrimidinase-like 3 | **↑** |
| Eph receptor B1 /// similar to Ndr3 protein /// similar to N-myc downstream regulated gene 3 | **↑** |
| Fibroblast growth factor receptor 1 | **↑** |
| glutamate receptor, ionotropic, N-methyl-D-aspartate 3A | **↑** |
| hairy and enhancer of split 1 (Drosophila) | **↑** |
| Inhibitor of DNA binding 3, dominant negative helix-loop-helix protein | **↓** |
| myelin basic protein | **↓** |
| nestin | **↓** |
| neurexin 1 | **↑** |
| neuroligin 1 | **↑** |
| reticulon 1 | **↑** |
| suppressor of cytokine signaling 2 | **↓** |
| transmembrane 4 superfamily member 11 | **↑** |
| ubiquitin carboxy-terminal hydrolase L1 | **↓** |
