# Supplementary material for: TGF-beta signalling in the adult neurogenic niche promotes stem cell quiescence as well as generation of new neurons
Source: J Cell Mol Med. 2014 Apr 30;18(7):1444–59. doi: 10.1111/jcmm.12298 (PMC4124027; doi:10.1111/jcmm.12298)
Supplement: Supplementary file 18 — Table S12. TGF-β1 regulated genes ‘cell fate determination’. [file jcmm0018-1444-SD18.doc]

| **Supp. Table 12.**  **TGF-beta1 regulated genes “cell fate determination”** | |
| --- | --- |
| **cell fate determination: z=1.42; p=0.157; fdr=0,189** | |
| gene title | regulation |
| achaete-scute complex homolog-like 1 (Drosophila) | **↑** |
| delta-like 1 (Drosophila) | **↑** |
| jagged 1 | **↑** |
