# Supplementary material for: TGF-beta signalling in the adult neurogenic niche promotes stem cell quiescence as well as generation of new neurons
Source: J Cell Mol Med. 2014 Apr 30;18(7):1444–59. doi: 10.1111/jcmm.12298 (PMC4124027; doi:10.1111/jcmm.12298)
Supplement: Supplementary file 19 — Table S12. TGF-β1 regulated genes ‘cell fate determination’. [file jcmm0018-1444-SD19.doc]

**Supplemental Material**

**Expression analysis of TGF-beta signaling components in the adult rat brain.**

We investigated protein expression of TGF-bRII, TGF-bRI, Smad2 and phosphorylation of Smad2 in the hippocampus (HC), ventricle wall including the subventricular zone (SVZ), olfactory bulb (OB), cortex (Cor) and cerebellum (CB) by Western blot analysis; lung tissue was used as control (Supp. Fig. 1). TGF-bRII and RI were detected in all brain regions with the neurogenic regions HC and SVZ showing the least expression. The highest expression of TGF-bRII was found in the lung. In contrast to that, the downstream signaling molecule Smad2 was strongly expressed in the different brain regions and only faintly detected in the lung. More importantly, while in lung tissue Smad2 was apparently in its non-phosphorylated and therefore inactive state, it was phosphorylated in the brain. This suggests that TGF-beta signaling is active in various regions of the adult rat brain including the neurogenic regions.

In order to substantiate the TGF-beta signaling profiling, we performed an extensive semiquantitative immunohistochemical analysis of the TGF-beta signaling components in different brain regions. Overall, we encountered a moderate to almost not detectable immunoreactivity of TGF-bRII in adult rat brain (Supp. Table 1). Faint perinuclear staining was present in cells of the CA1 and CA2 regions of hippocampus, in the cerebral cortex, in Purkinje cells of the cerebellum and cells of the brain stem (Supp. Table 1). Within the neurogenic regions TGF-bRII immunoreactivity was faint (Supp. Table 1). In contrast to the weak TGF-bRII immunoreactivity, a robust staining for TGF-bRI was noticed in the HC, in the rostramigratory stream (RMS) and in the OB. In the stem cell niche, expression of TGF-bRI was clearly visualized in the SGZ of HC and SVZ. In addition, expression of TGF-bRI was found in cells of the striatum, Cor, CB and in the brain stem (Supp. Table 1 and Supp. Fig. 2). A prevalent expression of pSmad2 was observed in most brain regions (Supp. Table 1 and Supp. Fig. 3). For example, pSmad2 immunoreactivity was prominent in Cor, striatum, CB and brain stem, in CA1, CA2 and CA3 regions of HC, in the SVZ, along the RMS and in the OB.

**Array analysis: Overall quantitative aspects**

As in our previous studies, where we described that TGF-beta1 inhibits NPC proliferation and promotes exit form the cell cycle, we stimulated adult rat hippocampus derived neurospheres for 7 days with 10 ng/ml TGF-beta1 or vehicle. RNA was then prepared and further processed for DNA array analysis in two independent experiments. First, we analyzed the overall reproducibility of the experiment and therefore compared the signal log ratios of the two experiments in a scatter-plot. The data and the resulting correlation coefficient of 0,81 indicates the vast majority of genes were regulated in a similar fashion in the two experiments. Overall, 872 probe-sets were significantly regulated by the TGF-beta1 stimulation with 448 probe-sets showing enhanced and 424 probe-sets showing a reduced gene expression (raw array data are accessible through GEO Series accession numbers GSE14562 and GPL1355 as well as GSE14556 and GPL341. This translated in 619 genes being regulated by TGF-beta1 with 248 (45,9%) of them being up-regulated and 335 (54,1%) being down-regulated. Supplementary Table 2 and 3 list the 100 genes that are down-, respectively up-regulated, the strongest. The functional assignments using the gene ontology category “biological function” illustrated that, among others, the most prevalent differential gene expression was present in the categories related to biosynthesis and metabolism, cell proliferation, cell growth, and cell cycle regulation, cell death and apoptosis, CNS development, neuronal maturation and synaptic transmission (Supplementary Table 5 – 12).

**Supplemetary Figures**

**Supp Figure 1**

**Western blot analysis demonstrating TGF-beta1 signaling components in intact adult brain.**

Western blots of brain homogenates were performed as described in Materal and Methods. Note the expressions of TGF-betaRII, TGF-betaRI and phosphorylated form of Smad2 in different brain regions. B-Brain, HC-Hippocampus, SVZ-Sub ventricular Zone, OB-Olfactory bulb, Cor-Cortex, CB-Cerebellum and non-neuronal tissue- Lung. Actin as control.

**Supp Figure 2**

**Localization of TGFb-RI immunoreactivity in different areas of intact adult rat brain.**

Immunehistochemistry of TGF-bRI was performed as described in Material and Methods. Note the widespread expression of TGF-bRI in DG-Dentate Gyrus, SVZ-Subventrivular Zone, OB-Olfactory bulb, Str-Striatum, Cor-Cortex, CB-Cerebellum and B Stem-brain stem. scale bar, 100µm. Insets are higher magnifications of selected fields.

**Supp Figure 3**

**Localization of pSmad2 immunoreactivity in different areas of intact adult rat brain.**

Immunehistochemistry of pSmad2 was performed as described in Material and Methods. Note the phosphorylated form of pSmad2 expression in DG-Dentate Gyrus, SVZ-Subventrivular Zone, OB-Olfactory bulb, Str-Striatum, Cor-Cortex, CB-Cerebellum and B Stem-brain stem. However, it is highly limited in stem cell niche, SGZ of DG and SVZ. scale bar, 100µm. Insets are higher magnifications of selected fields.

**Supp Fig 4**

**TGF-beta related signalling pathway analysis.**

The microarray data were analyzed to identify key regulators that control the expression of a variety of genes in NSCs upon TGF beta stimulation. A signalling related pathway analysis revealed expressions of TGFbeta receptors (indicated in Green) and R-Smads (Indicated in pink) as the most relevant downstream key molecules of TGF beta signaling which is indicated in the signalling flow chart.

**Supp Fig 5**

**TGF beta1 regulates expression of genes related to cell cycle of NSCs.**

Analysis of microarray data of TGF-beta1 treated vs vehicle treated neurospheres indicating changes the expression of genes that are involved in cell cycle control and indicated the expression of Mek1 (Map2k1) (indicated in pink) as a most prominent upstream molecule that is affected in NSCs by TGF beta treatment.

**Supp Fig 6**

**TGF-beta1 changes the gene expression of NeuroD and related molecules in NSCs.**

A pathway-related analysis on microarray data revealed that expressions of NeuroD (indicated in pink) and its pathway related genes are altered in NSCs treated with TGF-beta1.
